# Increased NBCn1 expression, Na^+^/${\text{HCO}}_{3}^{-}$ co-transport and intracellular pH in human vascular smooth muscle cells with a risk allele for hypertension

**DOI:** 10.1093/hmg/ddx015

**Published:** 2017-01-13

**Authors:** Fu Liang Ng, Ebbe Boedtkjer, Kate Witkowska, Meixia Ren, Ruoxin Zhang, Arthur Tucker, Christian Aalkjær, Mark J. Caulfield, Shu Ye

**Affiliations:** 1 Department of Clinical Pharmacology, William Harvey Research Institute, Barts and The London School of Medicine and Dentistry, Queen Mary University of London, London, UK; 2 Department of Biomedicine, Aarhus University, Aarhus, Denmark; 3 Department of Biomedicine, University of Copenhagen, Copenhagen, Denmark; 4 Department of Cardiovascular Sciences, University of Leicester, Leicester, UK; 5 NIHR Biomedical Research Centre in Cardiovascular Disease, Leicester, UK; 6 Shantou University Medical College, Shantou, China

## Abstract

Genome-wide association studies have revealed an association between variation at the *SLC4A7* locus and blood pressure. *SLC4A7* encodes the electroneutral Na^+^/${\text{HCO}}_{3}^{-}$ co-transporter NBCn1 which regulates intracellular pH (pH_*i*_). We conducted a functional study of variants at this locus in primary cultures of vascular smooth muscle and endothelial cells. In both cell types, we found genotype-dependent differences for rs13082711 in DNA-nuclear protein interactions, where the risk allele is associated with increased *SLC4A7* expression level, NBCn1 availability and function as reflected in elevated steady-state pH_*i*_ and accelerated recovery from intracellular acidosis. However, in the presence of Na^+^/H^+ ^exchange activity, the *SLC4A7* genotypic effect on net base uptake and steady-state pH_*i*_ persisted only in vascular smooth muscle cells but not endothelial cells. We found no discernable effect of the missense polymorphism resulting in the amino acid substitution Glu326Lys. The finding of a genotypic influence on *SLC4A7* expression and pH*_i_* regulation in vascular smooth muscle cells provides an insight into the molecular mechanism underlying the association of variation at the *SLC4A7* locus with blood pressure.

## Introduction

Hypertension is a common and a major etiological factor in cardiovascular, cerebrovascular and renovascular disease, estimated to cause up to 12.8% of global mortality and 3.7% of morbidity (1), with its complications contributing to a major worldwide economic burden (2). To compound the population health issue, up to 30% of patients with hypertension are inadequately treated (3), indicating a need for novel therapeutic approaches. There is a strong contribution from genes and their interaction with the environment to blood pressure (BP) regulation and the pathogenesis of hypertension (4,5). Recently, genome-wide association studies (GWAS) have identified a number of genomic loci at which common genetic variants have influences on BP (6–14). The study presented here attempts to translate the genetic information on a gene relevant to hypertension into a biomedical context. The identification of a path linking genetic variation to protein availability, protein activity and subsequently cellular behaviours provides further confidence in pursuing NBCn1 as a potential therapeutic target in hypertension.

One of the BP-associated loci identified by GWAS is on chromosome 3p24.1 encompassing the *SLC4A7* (solute carrier family 4 member 7, HGNC: 11033) gene (8). The lead GWAS single nucleotide polymorphism (SNP), rs13082711 (NC_000003.12:g.27496418T > C), at this locus showed an association with diastolic BP above the genome-wide significance threshold and a similar direction for systolic BP (*P* = 3.8 × 10^−^^9^ and 1.5 × 10^−^^6^, respectively) in a large-scale GWAS by the International Consortium for Blood Pressure (8). This SNP is in strong linkage disequilibrium with 92 other SNPs (LD, *r*^2 ^>^ ^0.8; Supplementary Material, Fig. S1A), with only one being a non-synonymous polymorphism (rs3755652; NC_000003.12:g.27431445C > T; Glu326Lys).

The *SLC4A7* gene encodes NBCn1, an electroneutral Na^+^/${\text{HCO}}_{3}^{-}$ co-transporter (15). At typical intracellular and extracellular ion concentrations, NBCn1 mediates electroneutral symport of Na^+ ^and ${\text{HCO}}_{3}^{-}$ into cells. Amongst cells expressing NBCn1 are those that play important roles in BP control, including vascular smooth muscle cells (VSMCs) (16–18), endothelial cells (VECs) (16,17,19) and epithelial cells of the medullary thick ascending limb of the loop of Henle (17,20,21). NBCn1 is known to have a role in controlling pH_*i*_ in VSMCs and VECs (18,19); and pH_*i*_ is a determinant of VSMC contractility (19,22–24) and endothelial function (19,23–26), both of which impact BP. In support, it has been shown that pH_*i*_ dysregulation in *SLC4A7* knockout mice results in an altered BP phenotype where the knockout mice show resistance to hypertensive stimuli such as angiotensin II, as compared to wildtypes (19).

In this study, we investigated if the BP-associated genetic variants at the *SLC4A7* locus identified by GWAS affect *SLC4A7* expression, NBCn1 protein availability and pH_*i*_ regulation in VSMCs and VECs. This is important for understanding the functional effects of these genetic variants and the mechanisms associated with their influence on BP.

## Results

### Allelic difference in *SLC4A7* expression level

**Figure 1:**
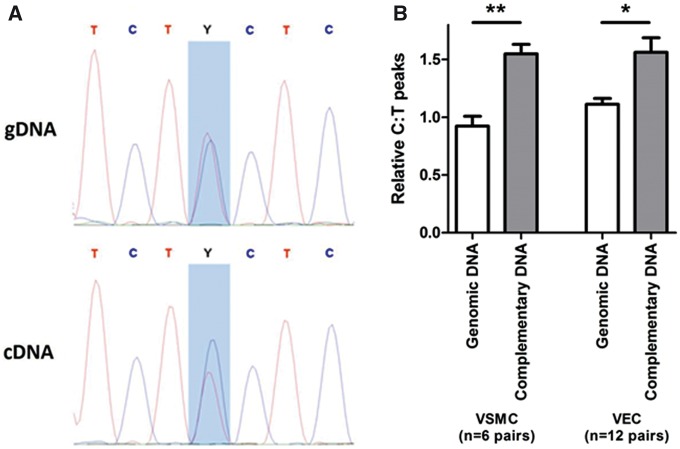
The BP risk (minor) allele at the *SLC4A7* locus is associated with increased gene expression. **(A)** Allelic imbalance analyses using heterozygotes at an exonic SNP (rs13096477), which is in high LD with the GWAS lead SNP (rs13082711). Representative chromatographs of genomic DNA (gDNA) and complementary DNA (cDNA) for the same sample, with the C/T SNP rs13096477 highlighted in blue, which is quantified in **(B)**, where the C (BP-risk) allele is preferentially observed in cDNA of VSMC and VEC samples. *P<0.05, **P<0.01, by paired *t*-test.

The lead BP GWAS SNP rs13082711 is in high LD (*r*^2 ^≥^ ^0.8) with 92 other SNPs which together span a 134 kb genomic interval. *SLC4A7* is the only gene located within this interval (Supplementary Material, Fig. S1A) and the genes closest on either side are *NEK10* (NIMA-related kinase 10, HGNC: 18592) and *EOMES* (eomesodermin, HGNC: 3372) residing 255 and 232 kilobases, respectively, away from the lead BP SNP. To investigate if the BP-associated variants influence the expression of *SLC4A7*, *NEK10* and/or *EOMES*, we first performed RT-PCR assays of these genes to determine if they were expressed in VSMCs and/or VECs. These assays showed that *SLC4A7* was consistently expressed in both cell types, whereas neither *NEK10* nor *EOMES* was expressed in either cell type (Supplementary Material, Fig. S1B). Having found that *SLC4A7* was expressed, but both *NEK10* and *EOMES* were not readily detected in VSMCs and VECs, we conducted subsequent experiments focusing on *SLC4A7*, starting with allelic expression imbalance analyses. Since this technique entailed the analysis of a SNP in the coding region, we analyzed the non-synonymous coding SNP rs13096477 (NC_000003.12:g.27448703T > C) which is in strong LD (*r*^2 ^>^ ^0.95) with the BP GWAS index SNP rs13082711 located upstream of the gene and therefore not directly analyzed in this assay. The analyses showed that in both VSMCs and VECs, *SLC4A7* RNA expression level of the BP-raising (minor, C) allele at rs13096477 was higher as compared to that of the alternative (major, T) allele (Fig. 1A and B).

To investigate the potential molecular mechanism leading to the allelic difference in *SLC4A7* RNA level described above, we ascertained whether there was an allelic effect on DNA-nuclear protein interaction, an important aspect in gene transcriptional regulation. To this end, we undertook formaldehyde-associated isolation of regulatory elements (FAIRE) studies coupled with allelic imbalance assay to investigate if there was an allelic-dependent difference in nuclear protein binding in the intact cellular environment. Since this technique required analysis of an intragenic SNP, as for the allelic expression imbalance assay described above, we again analyzed the SNP rs13096477. The analysis showed an allelic imbalance of rs13096477, with its minor (BP-elevating, T) allele being in preferentially open chromatin conformation in VSMCs (Supplementary Material, Fig. S2A). This allelic imbalance in DNA-protein binding was not detected in VECs.

**Figure 2:**
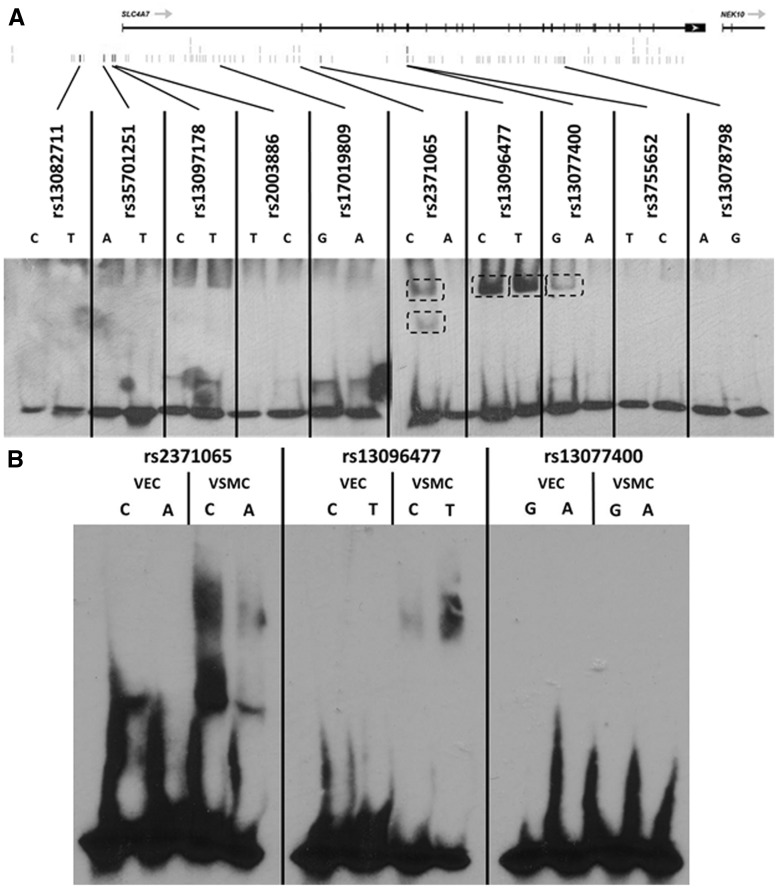
The *SLC4A7* BP-associated locus shows allele-dependent DNA-nuclear protein interactions. **(A)** EMSA with labelled-probes corresponding to both alleles of the 10 highest priority SNPs at *SLC4A7*. The start codon *NEK10* appears on the right or of the figure. Unbound probes as visualized at bottom of autoradiograph. Representative figure of *n*=3 replicates. **(B)** EMSA comparing VEC and VSMC nuclear extracts for three SNPs shows the shift present in VSMC nuclear extracts virtually absent with VEC nuclear extracts. Representative figure of *n*=2 replicates. All lanes had 10 femtomoles of labelled double-stranded oligonucleotide probe with 10 ng of corresponding nuclear extracts.

Since rs13096477 and rs13082711 are in high LD (*r*^2 ^≥^ ^0.8) with 92 other SNPs, and the FAIRE allelic imbalance assay technique could not provide information about which of these SNPs in high LD was responsible for the allelic imbalance results described above, we carried out electrophoretic mobility shift assays (EMSA) on 10 selected SNPs among the 93 in high LD, prioritized based on their positions relative to the start of *SLC4A7* transcription and transcription factor binding site predictions from various bioinformatics resources including ENCODE and RegulomeDB (Supplementary Material, Fig. S2B). EMSAs on the 10 tested SNPs showed VSMC nuclear protein binding preferentially to the major allele of rs13096477 but, the minor allele of rs2371065 (NC_000003.12:g.27453098A > C), and weakly to the minor allele of rs13077400 (NC_000003.12:g.27431575A > G) (Fig. 2A, Supplementary Material, Fig. S3A–C). In contrast, neither allele of the BP GWAS index SNP rs13082711 showed DNA-protein interaction in EMSAs. Consistent with the FAIRE results mentioned above, EMSAs with VEC nuclear protein extracts did not detect binding with any of the DNA probes for the 10 tested SNPs (Fig. 2B).

Since the above EMSAs showed allele-specific binding of a nuclear protein to the rs2371065 minor allele, we undertook further experiments in an attempt to determine the identity of this nuclear protein. We performed supershift assays with antibodies against the nuclear proteins PHOX2A, PLAG1 and TFAP2C, respectively, as bioinformatics analyses showed that their recognition DNA sequences had some similarities with the DNA sequence encompassing the rs2371065 site. However, these assays show no evidence to suggest that any of these proteins being the one interacting with the rs2371065 minor allele shown in EMSAs (Supplementary Material, Fig. S4). We next conducted a DNA pull-down assay using a biotin-labelled double-stranded oligonucleotide corresponding to the DNA sequence at and surrounding the site of the rs2371065 minor allele. An electrophoretic analysis of proteins pulled down by this oligonucleotide showed two bands consistent with the EMSA results described earlier, neither of which was present in the negative control (Supplementary Material, Fig. S5). However, *N*-terminal protein sequencing failed to reveal the identity of the protein(s) pulled down by the oligonucleotide.

### Allelic difference in NBCn1 protein level

**Figure 3:**
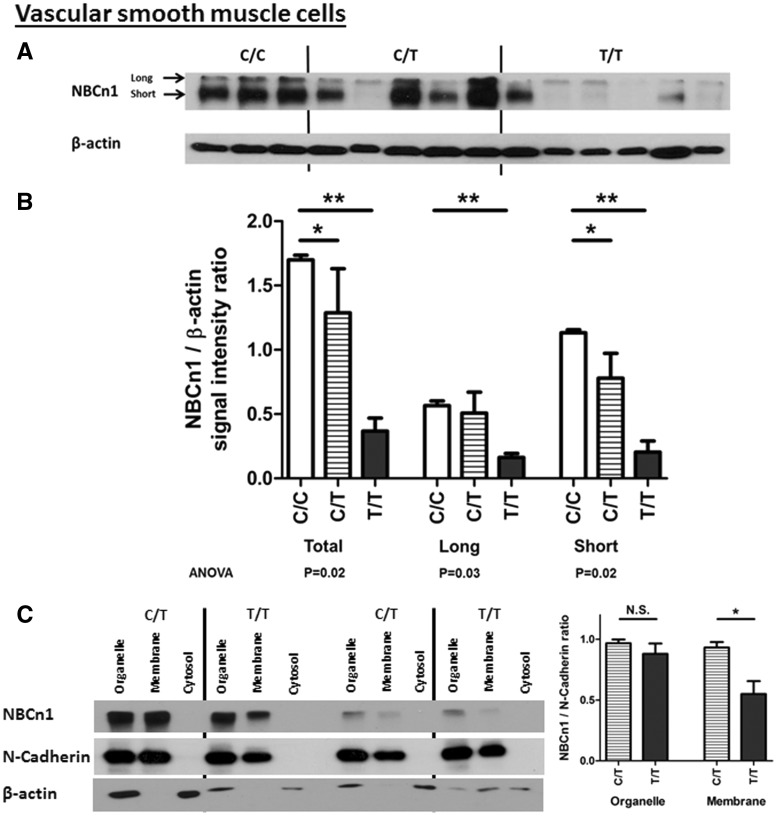
The BP risk (minor) allele at the *SLC4A7* locus is associated with increased NBCn1 protein expression and availability at the plasma membrane in VSMCs. **(A)** Protein immunoblots for NBCn1 with VSMC samples stratified by genotype at rs13082711, where the two signals for NBCn1 reflecting the isoforms with and without Cassette II (band sizes approximately 150 kDa and 135 kDa, respectively), quantified in **(B)**, where comparisons across the three genotypes show increased protein expression for risk allele carriers. *P<0.05, by one-way ANOVA after Bonferroni correction for multiple comparisons. **(C)** Subcellular fractionation samples from VSMCs, showing that the BP risk (minor) allele at the *SLC4A7* locus is associated with increased NBCn1 signal in the membrane fraction. 2 μg protein of each cellular fraction was loaded in each lane. *n*=9 in each group *P<0.05 by Mann-Whitney *U*-test.

**Figure 4:**
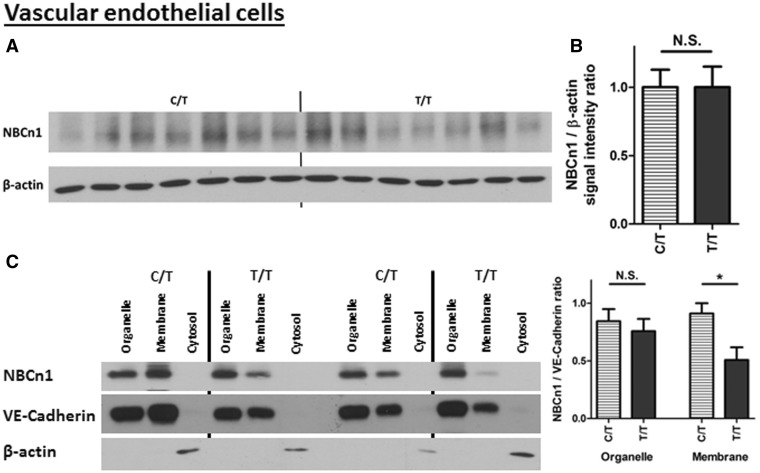
The BP risk (minor) allele at the *SLC4A7* locus is associated with increased NBCn1 availability at the plasma membrane in VECs. **(A)** shows the protein immunoblots for NBCn1 with VEC samples stratified by genotype at rs13082711, and quantified in **(B)** P > 0.05 by Mann-Whitney *U*-test. **(C)** Subcellular fractionation samples from VECs, showing that the BP risk (minor) allele at the *SLC4A7* locus is associated with increased NBCn1 signal in the membrane fraction. 2 μg protein of each cellular fraction was loaded in each lane. *n*=8 each group. *P<0.05 by Mann-Whitney *U*-test.

Having found an allelic difference in *SLC4A7* RNA expression level as described above, we investigated if there was a corresponding difference in NBCn1 protein level. Immunoblot analyses of VSMC total cellular protein extracts showed that NBCn1 protein levels were the greatest in minor (BP-raising) allele homozygotes, intermediate in heterozygotes and lowest in major allele homozygotes (Fig. 3A and B). Despite the allelic imbalance in RNA/cDNA levels, there were no detected genotypic differences in VEC total cellular NBCn1 protein expression (Fig. 4A and B).

Following the above analysis of total cellular NBCn1, we examined NBCn1 in subcellular locations, more specifically, their presence at the cellular membrane. An immunoblot analysis of subcellular protein fractionations prepared by differential centrifugation showed that the cells carrying the BP-raising allele (C/T genotype for rs13082711) had higher levels of NBCn1 in the membrane fraction as compared to non-carriers (T/T genotype) in both VSMCs (Fig. 3C) and VECs (Fig. 4C). The vesicle and organelle fraction also contained NBCn1 but the difference between the genotypes was less pronounced. The supernatant (cytosolic) fraction had no detectable expression of NBCn1 or the plasma membrane markers N-cadherin and VE-cadherin.

### Association of the BP-raising allele with increased Na^+^/${\text{HCO}}_{3}^{-}$ co-transport activity in VSMCs, which is not overcome by Na^+^/H^+ ^exchange activity

**Figure 5:**
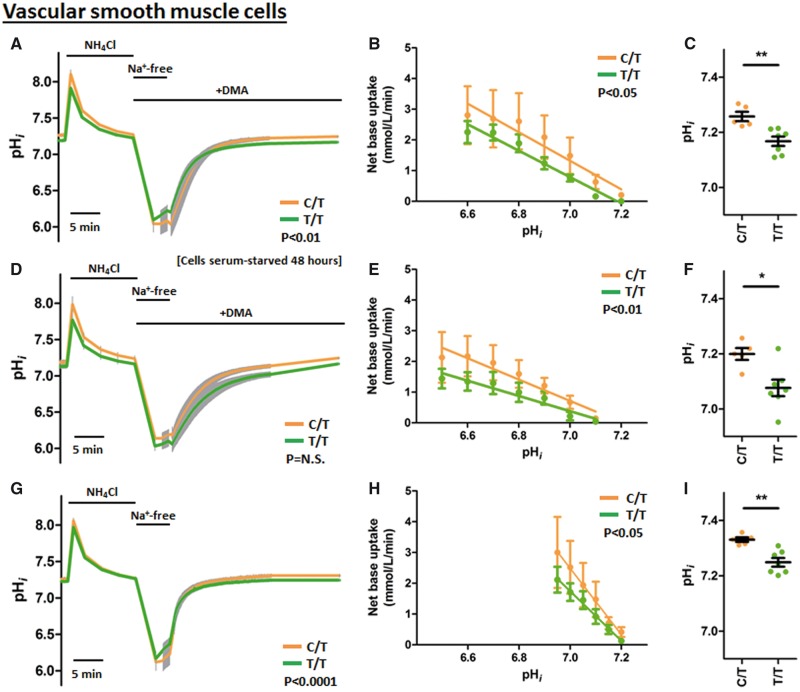
The BP risk (minor) allele at the *SLC4A7* locus is associated with increased Na^+^/${\text{HCO}}_{3}^{-}$ co-transport activity in VSMCs and is not overcome by Na^+^/H^+ ^exchange activity. Intracellular pH recovery of VSMCs following ${\text{NH}}_{4}^{\text{+}}$-prepulse-induced intracellular acidosis. Experiments were performed with **(A–C)** CO_2_/${\text{HCO}}_{3}^{-}$ and 30 µM DMA, **(D–F)** CO_2_/${\text{HCO}}_{3}^{-}$ and 30 µM DMA, in cell cultures serum-starved for 48 h beforehand, and **(G–I)** in CO_2_/${\text{HCO}}_{3}^{-}$ conditions. The risk allele carriers (orange, C/T at rs13082711, *n*=5) had faster pH_*i*_ recovery and higher final plateau pH_*i*_ compared to protective allele homozygotes (green, T/T at rs13082711, *n*=7), even in the absence of DMA. (A,D,G) Intracellular pH traces of VSMCs during ammonium prepulse studies. Grey error bars denotes SEM. Periods of exposure to 20 mM NH_4_Cl, Na^+^-free buffers, or 30 µM DMA are marked above the traces. X-axis scale bar denotes 5 min. Groups compared by repeated-measures two-way ANOVA over the 280 time points between 60 s and 900 s after reintroduction of Na^+^-containing buffer. (B,E,H) Net base uptake for each group calculated at specified pH_*i*_ values. Slopes compared by least-squares linear regression analyses. (C,F,I) Final plateau pH_*i*_ after recovery from intracellular acidosis. *P<0.05, **P<0.01 by Mann–Whitney *U*-test.

To determine whether the increased *SLC4A7* gene expression and NBCn1 protein availability at the plasma membrane in cells carrying the BP-raising allele gives rise to increased protein function, the capacity of these primary cultured cells for pH_*i*_ recovery from ${\text{NH}}_{4}^{\text{+}}$-prepulse-induced intracellular acidification was assessed. Comparing VSMCs from BP-raising allele carriers (C/T risk allele carriers at rs13082711, *n =* 5) and protective allele homozygotes (T/T genotype, *n =* 7), we observed a difference in pH_*i*_ recovery rate following intracellular acidosis in cells exposed to the Na^+^/H^+ ^exchange inhibitor dimethylamiloride (DMA, 30 µM) in the presence of CO_2_/${\text{HCO}}_{3}^{-}$ (Fig. 5A). As the activities of acid-base transporters are regulated by pH_*i*_, the rate of net base uptake was determined at regular pH_*i*_ intervals (Fig. 5B), demonstrating a genotype effect, where VSMCs from the rs13082711 BP-raising allele carriers had a higher rate of net base uptake at each pH_*i*_ compared to protective allele homozygotes. In addition to differences in net base uptake rates, there was also a genotype effect on final plateau pH_*i*_ that was revealed in the presence of DMA (ΔpH_*i *_=_* *_0.087 ± 0.027, *P* < 0.01) (Fig. 5C). This observed genetic influence was confirmed to be CO_2_/${\text{HCO}}_{3}^{-}$-dependent as the difference in net base uptake and final resting pH*_i_*disappears in CO_2_/${\text{HCO}}_{3}^{-}$-free conditions (Supplementary Material, Fig. S6A–C).

To minimize the potential effects of variances in cell cycles, the VSMCs were also tested after 48 h of serum-starvation (27). In this synchronized state, the Na^+^- and CO_2_/${\text{HCO}}_{3}^{-}$-dependent, DMA-insensitive pH_*i*_ recovery and the final plateau pH_*i*_ were lower than under serum-stimulated conditions but still higher in BP-raising allele carriers compared to cells homozygous for the protective allele (Fig. 5D–F).

While these data show a genetic influence on Na^+^/${\text{HCO}}_{3}^{-}$ co-transport activity in the absence of Na^+^/H^+ ^exchange activity (inhibited by DMA), it does not necessarily indicate an overall effect on pH_*i*_ under physiological conditions where Na^+^/H^+ ^exchange may play a large role. It is therefore important to note that in the presence of Na^+^/H^+ ^exchange activity (i.e. in the absence of DMA), the genotype-associated difference in net base uptake and final plateau pH_*i*_ persisted (Fig. 5G–I).

As expected, the buffering capacity of the VSMCs was higher in the presence of CO_2_/${\text{HCO}}_{3}^{-}$ than in its nominal absence. This was particularly evident at the pH_*i*_ ranges closer to physiological levels where [${\text{HCO}}_{3}^{-}$]_*i*_ is high and adds substantially to the buffering power (Supplementary Material, Fig. S7A). There were no differences in buffering capacity between cells of the two assessed genotypes (Supplementary Material, Fig. S7B) in the presence or absence of CO_2_/${\text{HCO}}_{3}^{-}$.

### Association of the BP-raising allele with increased NBCn1 activity in VECs, which is overcome by Na^+^/H^+ ^exchange activity

**Figure 6:**
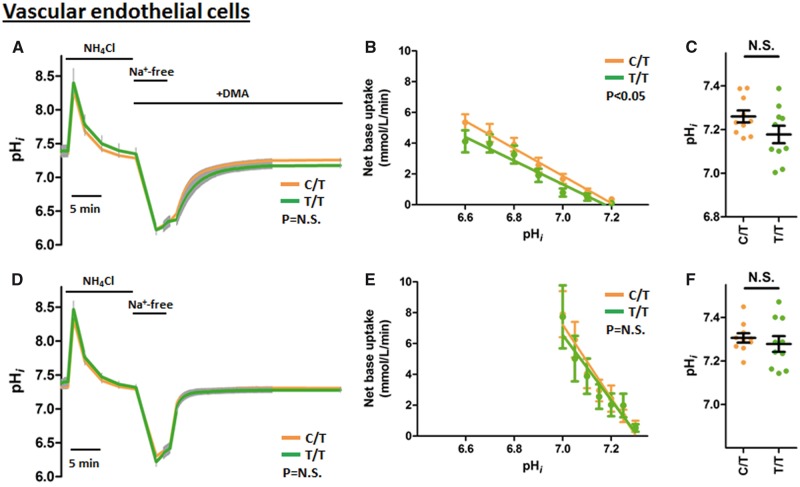
The BP risk (minor) allele at the *SLC4A7* locus is associated with increased Na^+^/${\text{HCO}}_{3}^{-}$ co-transport activity in endothelial cells but it is masked by Na^+^/H^+ ^exchange activity. Intracellular pH recovery of VECs following ${\text{NH}}_{4}^{\text{+}}$-prepulse-induced intracellular acidosis. Experiments were performed with (**A–C**) CO_2_/${\text{HCO}}_{3}^{-}$ and 30 µM DMA and, (**D–F**) in CO_2_/${\text{HCO}}_{3}^{-}$ conditions. The risk allele carriers (orange, C/T at rs13082711, *n*=10) had faster pH_*i*_ recovery and higher final plateau pH_*i*_ compared to protective allele homozygotes (green, T/T at rs13082711, *n*=10), but this difference was abolished in the absence of DMA. (A and D) Intracellular pH traces of VECs during ammonium prepulse studies. Grey error bars denotes SEM. Periods of exposure to 20 mM NH_4_Cl, Na^+^-free buffers or 30 µM DMA are marked above the traces. X-axis scale bar denotes 5 min. Groups compared by repeated-measures two-way ANOVA over the 280 time points between 60 s and 900 s after reintroduction of Na^+^-containing buffer. (B and E) Net base uptake for each group calculated at specified pH_*i*_ values. Slopes compared by least-squares linear regression analyses. (C and F) Final plateau pH_*i*_ after recovery from intracellular acidosis.

In a study of VEC samples from BP-raising allele carriers (C/T genotype at rs13082711, *n =* 10) and protective allele homozygotes (T/T genotype, *n =* 10), no significant difference in pH_*i*_ recovery following intracellular acidosis was detected by repeated measures two-way ANOVA (Fig. 6A) despite the apparent divergence of the curves in cells exposed to Na^+^/H^+ ^exchange inhibition (30 µM DMA) in the presence of CO_2_/${\text{HCO}}_{3}^{-}$. When calculated and plotted as a function of pH_*i*_, this revealed a genotype effect on the rate of net base uptake (Fig. 6B). Similar to VSMCs, there was a higher final plateau pH_i_ that was revealed in the presence of DMA for C/T risk allele carriers compared to T/T protective allele homozygotes (ΔpH_*i *_=_* *_0.082 ± 0.053, *P* = 0.10, Fig. 6C). This is a similar magnitude to that of VSMCs, but due to the larger confidence interval, did not reach statistical significance. Similarly, there was no significant difference in buffering capacities between the genotypes of VECs when compared in the pH_*i*_ range covered by both the C/T and T/T groups (Supplementary Material, Fig. S7C and D). Once again, the observed genetic influence on net base uptake was confirmed to be CO_2_/${\text{HCO}}_{3}^{-}$-dependent with its disappearance in CO_2_/${\text{HCO}}_{3}^{-}$-free conditions (Supplementary Material, Fig. S6D–F).

Similar to the analysis of VSMCs above, a more physiological condition with uninhibited Na^+^/H^+ ^exchange needed to be considered. In this setting, unlike that of VSMCs, the genotype-associated difference in VEC net base uptake rate and final plateau pH_*i*_ was overcome by the presence of Na^+^/H^+ ^exchange activity (Fig. 6D–F). Taken in combination, this may indicate that the BP-associated locus is more likely to exert its effects via VSMCs rather than VECs.

### Bioinformatic tools predict the NBCn1 Glu326Lys variation to be well tolerated

Having identified that the BP-raising allele (C for rs13082711) is associated with higher Na^+^/${\text{HCO}}_{3}^{-}$ co-transport activity and steady-state resting pH_i_, we wondered whether the missense Glu326Lys amino acid change (SNP at rs3755652) in high LD with rs13082711 (*r*^2 ^>^ ^0.9) contributes to this effect. The main difference between glutamic acid and lysine amino acid residues is that the former has a negative charge; whist the latter has a positive charge. They are otherwise relatively similar - having similar molecular weights, both being hydrophilic, and neither is bulky enough to result in steric hindrance or changes in secondary structures. This similarity is reflected by the early analysis by Grantham (28), noting that the functional effect of this amino acid change is predicted to be relatively small. Utilizing seven other online prediction tools, the overall consensus was that the Glu326Lys is a well-tolerated amino acid change (Supplementary Material, Table S1).

### The NBCn1 Glu326Lys variant does not affect NBCn1 activity

**Figure 7:**
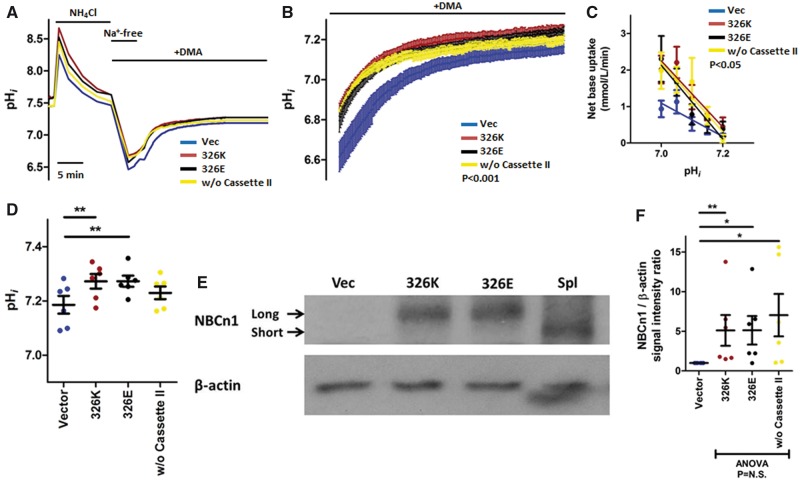
The Glu326Lys amino acid variation at the BP risk locus does not influence NBCn1 activity. Intracellular pH recovery of A10 cells transfected with *SLC4A7*-expression plasmids (pcDNA) following ${\text{NH}}_{4}^{\text{+}}$-prepulse-induced intracellular acidosis. Experiments were performed with CO_2_/${\text{HCO}}_{3}^{-}$ and 30 µM DMA*.* Blue denotes Vector; Brown, 326K (lysine); Black, 326E (glutamic acid); Yellow, without Cassette II. Transfection with any of the three expression plasmids increased pH_*i*_ recovery and final plateau pH_*i*_ compared to vector control (*n*=6 sets). (**A**) Intracellular pH traces of A10 cells transfected with *SLC4A7*-expression plasmids during ammonium prepulse studies. Periods of exposure to 20 mM NH_4_Cl, Na^+^-free buffers or 30 µM DMA are marked above the traces. X-axis scale bar denotes 5 min. Groups compared by repeated-measures two-way ANOVA over the 280 time points between 60 and 900 s after reintroduction of Na^+^-containing buffer. (**B**) shows the zoomed in section between time points 0 and 600 s after reintroduction of Na^+^-containing buffer. Error bars denotes SEM. Groups compared by repeated-measures two-way ANOVA. (**C**) Net base uptake for each group calculated at specified pH_*i*_ values. Slopes compared by least-squares linear regression analyses. (**D**) Final plateau pH_*i*_ after recovery from intracellular acidosis. *P*<0.01, by repeated-measures one-way ANOVA; **P<0.01, by paired *t*-test after Bonferroni correction. (**E**) Representative immunoblot of samples after transfection with vector or overexpression plasmids, quantified in (**F**), where the cells transfected with overexpression plasmids had higher expression levels compared to vector control, *P<0.05, by paired *t*-test after Bonferroni correction. There were no differences when the three overexpression studies were compared to each other by repeated-measures one-way ANOVA.

To assess the hypothesis that the Glu326Lys variant does not affect NBCn1 activity, we overexpressed the 326Glu and 326Lys variants and a shorter variant that lacks splice Cassette II (amino acids 251–374, thus lacking the Glu326Lys variation) of NBCn1 in A10 cells, and assessed their capacity for pH_*i*_ recovery following ${\text{NH}}_{4}^{\text{+}}$-prepulse-induced intracellular acidosis in the presence of CO_2_/${\text{HCO}}_{3}^{-}$ and DMA. Cells transfected with any of the three overexpression plasmids displayed faster pH_*i*_ recovery from intracellular acidification compared to cells transfected with the control vector, but there were no differences between the impact of the three overexpression plasmids (Fig. 7A and B). This was verified in the analysis accounting for net base uptake rate as a function of pH_*i*_ (Fig. 7C). In addition to the increased pH_*i*_ recovery rate, A10 cells transfected with overexpression plasmids also have a higher plateau pH_*i*_ as compared to cells transfected with the control vector, but again, there were no differences between cells transfected with the three overexpression plasmids (Fig. 7D). These findings were obtained with consistent and comparable overall overexpression across the three different NBCn1 variants (Fig. 7E and F), and similar to primary VSMCs and VECs observed before, there was no difference in buffering capacities (Supplementary Material, Fig. S7E). These findings suggest that the Glu326Lys amino acid variation does not alter the intrinsic acid-base transport activity of NBCn1, consistent with the aforementioned bioinformatics predictions.

### Calcineurin inhibition did not influence Na^+^/${\text{HCO}}_{3}^{-}$ co-transport activity under the experimental conditions

Although there is evidence that NBCn1 activity is regulated by phosphorylation, perhaps via calcineurin (29,30), the absence of an effect of either the Glu326Lys amino acid variation, and the variant that lacks Cassette II (Fig. 7), led us to postulate that the genotypic effect was independent of calcineurin activity. Furthermore, the genotypic effect was observed in conditions with lower intracellular [Ca^2+^], where calcineurin is less likely to play a role (30). To assess this hypothesis, primary VSMCs were assessed for Na^+^- and CO_2_/${\text{HCO}}_{3}^{-}$-dependent, DMA-insensitive pH_*i*_ recovery rate following ${\text{NH}}_{4}^{\text{+}}$-prepulse-induced intracellular acidosis while being exposed to 10 µM of the calcineurin inhibitor FK506 (or equivolume DMSO vehicle). In these conditions, we did not observe an effect of 10 µM FK506 on DMA-insensitive Na^+^/${\text{HCO}}_{3}^{-}$ co-transport activity (Supplementary Fig. S8A and B) and it stands to follow that even in the presence of 10 µM FK506, the genotype-associated difference in DMA-insensitive Na^+^/${\text{HCO}}_{3}^{-}$ co-transport activity persists (Supplementary Material, Fig. S8C and D).

To confirm the above findings from primary VSMCs, A10 cells transfected with the three different overexpression plasmids (326Lys, 326Glu and the variant without splice cassette II) were also investigated under the same conditions. Consistent with the findings from primary VSMCs, A10 cells transfected with the different overexpression plasmids continues to show the lack of effect of calcineurin inhibition by 10 µM FK506 on Na^+^/${\text{HCO}}_{3}^{-}$ co-transport activity (Supplementary Material, Fig. S8E and F). Again these findings are consistent with previous reports that calcineurin inhibition only affects NBCn1 activity under conditions of elevated intracellular [Ca^2+^] (30).

## Discussion

We here identify a potential mechanism for the impact of the *SLC4A7* BP-associated locus on vascular cells. Mechanistically, our studies demonstrate allele-associated differences in DNA-nuclear protein interaction, gene expression and NBCn1 function, where the carriers of the *SLC4A7* BP risk allele demonstrated increased NBCn1 protein expression, and in turn, upregulated net base uptake rate and higher steady-state pH_*i*_. Although the increased expression and NBCn1 activity are also found in VECs, the effect on net acid extrusion rate and steady state pH_*i*_ was masked by a larger Na^+^/H^+ ^exchange (DMA-sensitive) activity under our experimental conditions.

GWAS have identified multiple genetic loci associated with BP, each with a modest contribution to overall BP levels. One of these was found to encompass *SLC4A7/*NBCn1. NBCn1 regulates pH_*i*_ by electroneutral symport of Na^+ ^and ${\text{HCO}}_{3}^{-}$ into cells. Reduction of NBCn1 activity in VSMCs and VECs, by knockdown (18) or knockout (19), abolishes Na^+^/${\text{HCO}}_{3}^{-}$ co-transport and markedly attenuates pH_*i*_ recovery from intracellular acidosis. As there appears to be opposing effects of altered NBCn1-function in VSMCs and VECs on BP demonstrated by the *SLC4A7* knockout mouse (19), it is important to identify in which cell type, if any, NBCn1 allele variation has an effect.

The role of vascular pH_*i*_ regulation in human hypertension is supported by the finding that vascular segments from hypertensive patients are more resistant to noradrenaline-induced intracellular acidification than segments from normotensive controls (31). That study was conducted prior to in-depth characterization of Na^+^/${\text{HCO}}_{3}^{-}$ co-transporters, and more recent reports show that NBCn1-mediated Na^+^/${\text{HCO}}_{3}^{-}$ co-transport has subsequently been shown to protect VSMCs against intracellular acidification during contractions (18,30,32). As intracellular acidification of VSMCs lowers rho-kinase-dependent VSMC Ca^2+ ^sensitivity (19,23), the improved ability to eliminate the contraction-induced intracellular acid load may contribute to the higher peripheral arterial resistance of hypertensive patients through increased rho-kinase activity. Consistent with the effect of sustained intracellular acidification on VSMC Ca^2+ ^sensitivity, knockout of NBCn1 lowers noradrenaline-induced contractions of mesenteric arteries (19) and myogenic responses of pressurized middle cerebral arteries (24) after endothelial blockade without affecting VSMC membrane potential or [Ca^2+^]_*i*_. There is also evidence that changes in acid-base transport function and/or pH_*i*_ impact *in vitro* VSMC proliferation (33), migration and viability (34), and medial wall thickness (23). NBCn1 plays a key role for VSMC migration and carotid artery remodeling most likely because it establishes local pH_*i*_ gradients and promotes filopodia, which can explain the decelerated directional migration of VSMCs from NBCn1 knockout mice (35). Altered NBCn1 activity could therefore modify vascular remodeling with long-term impact on peripheral resistance.

Endothelial function is impaired by intracellular acidosis (23,25); and endothelial NO production is reduced in *SLC4A7* knockout mice without any change in endothelial [Ca^2+^]_*i*_ or NO synthase expression (19). Intracellular pH has also been shown to influence the generation of endothelial vasoactive substances such as the expression of endothelin (26) and the intrinsic activity of nitric oxide synthase (25). However, the disappearance of the genotypic effect in VECs when Na^+^/H^+ ^exchange is present suggests that the GWAS identified genetic variance is unlikely to exert its effect through endothelial function. This is with the caveat of these studies being conducted in an *in vitro* system, whereas *in vivo*, endothelial cells would be exposed to shear stress as well as circulating hormonal factors.

**Figure 8:**
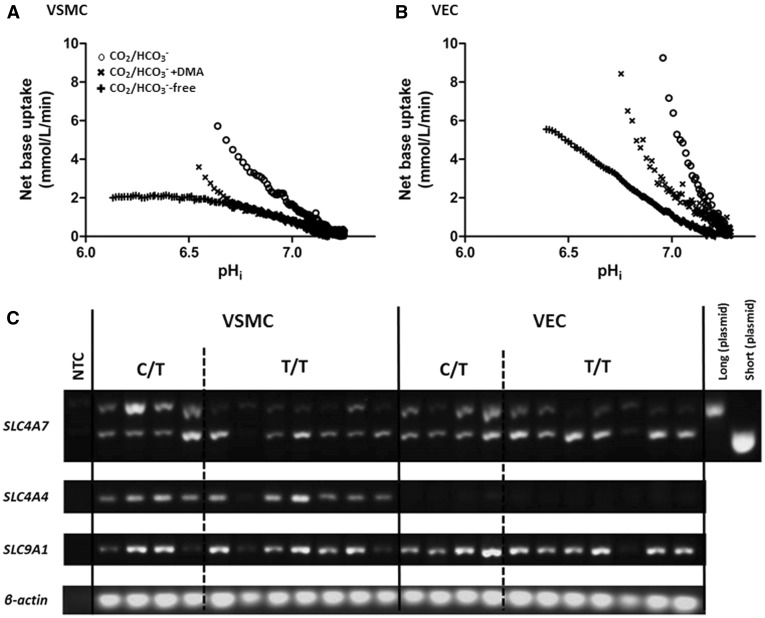
Na^+^/${\text{HCO}}_{3}^{-}$ co-transport and Na^+^/H^+ ^exchange activities are similar in VSMCs, but Na^+^/H^+ ^exchange dominates in VECs. Average net base uptake plotted against average pH_*i*_ for each corresponding time-point after ${\text{NH}}_{4}^{\text{+}}$-prepulse-induced intracellular acidosis of (**A**) VSMCs (*n*=12) and (**B**) VECs (*n*=20) in the presence and absence of either 30 µM DMA or CO_2_/${\text{HCO}}_{3}^{-}$. Open circles denote studies with CO_2_/${\text{HCO}}_{3}^{-}$; cross, CO_2_/${\text{HCO}}_{3}^{-}$ + DMA; plus, CO_2_/${\text{HCO}}_{3}^{-}$-free conditions. (**C**) shows result of end-point RT-PCR (10 ng of reverse-transcribed RNA) of selected pH_*i*_ regulators (long and short variants of *SLC4A7*, together with other pH_i_ regulators *SLC4A4* (NBCe1) and *SLC9A1* (NHE1)) in paired samples of VSMCs and VECs, showing significant inter-sample variability. β-actin included as positive control.

We found that VECs have a higher level of *SLC4A7* expression and faster Na^+^- and CO_2_/${\text{HCO}}_{3}^{-}$-dependent, DMA-insensitive recovery from intracellular acidosis as compared to VSMCs (Supplementary Material, Fig. S9). Importantly, the genotype-associated differences found in Na^+^/${\text{HCO}}_{3}^{-}$ co-transport activity are masked by Na^+^/H^+ ^exchange in VECs, but not VSMCs. This may be related to the relative contributions of Na^+^/H^+ ^exchange and Na^+^/${\text{HCO}}_{3}^{-}$ co-transport being similar to each other in VSMCs, especially in the pH_*i*_ ranges from around 6.7 upwards (Fig. 8A). This would enable subtle genotype-associated differences in NBCn1 activity to persist in VSMCs, but not in VECs where Na^+^/H^+ ^exchange contribution far outweighs that of Na^+^/${\text{HCO}}_{3}^{-}$ co-transport (Fig. 8B). These results should be taken in relation to the expression of other pH_*i*_ regulators (Fig. 8C), where a panel of paired VSMC and VEC samples shows inter-sample variability with *SLC4A7* short and long isoforms (confirming the previous qRT-PCR and immunoblot results), but also *SLC4A4* (NBCe1) and *SLC9A1* (NHE1). Although mRNA expression of multiple *SLC4*-family Na^+^/${\text{HCO}}_{3}^{-}$ co-transporters has also been identified in mouse carotid arteries, NBCn1 has been found to functionally dominate net acid extrusion (35). Notably, we found a minimal signal for *SLC4A4* in VECs.

The genotype-associated changes in overall VSMC pH_*i*_ regulation may be related not just to resting pH_*i*_, but also to the rate from which pH_*i*_ recovers from intracellular acidosis; particularly as pH_*i*_ is not constant and pH_*i*_ recovery rates are important in response to a variety of *in vivo* vasoconstrictive stimuli such as angiotensin II, noradrenaline, endothelin-1 and cellular depolarization (31,36–38). It is hitherto unknown whether it is the resting steady-state pH_*i*_, or the ability to return towards its set-point and avoid intracellular acidification during contractions that predominantly influences the overall *in vivo* phenotype but our results show that *both* the rate of recovery and the resting levels of pH_*i*_ are influenced by *SLC4A7* genotype.

The direction of effect for the data presented here is congruent with that of the *SLC4A7* knockout model (19); where the knockout mice are protected from the hypertensive stressor angiotensin II, we show that the BP-protective allele is associated with the reduced NBCn1 expression and slower recovery from intracellular acidosis, particularly in VSMCs. The clinical relevance of the described genotypic effects is supported by observational data from arterial segments from humans with hypertension: in human resistance arteries from hypertensive subjects, the VSMCs were protected from intracellular acidosis after noradrenaline contraction relative to those from normotensive subjects (31). Additionally, when intracellular acidosis was induced in mouse mesenteric artery studies, a fall of VSMC pH_*i*_ by approximately 0.1 was associated with a decreased *ex vivo* contractility (19). The differences in resting pH*_i_* (95% CI, 0.08–0.12) and pH*_i_* recovery rates (95% CI, 15–55%) that we describe between the *SLC4A7* genotypes – with both parameters higher in rs13082711 risk allele carriers as compared to the protective allele homozygotes – may be clinically significant if they continue to persist *in vivo*.

A recently reported study shows that SNP rs820430, which is located in an intergenic region ∼23 kb from *SLC4A7* and associated with BP in a Chinese Han population GWAS (39), has an effect on *SLC4A7* transcription in peripheral blood mononuclear cells (40). In the present study, we investigated a separate genetic signal represented by rs13082711, the lead BP-associated SNP in GWASs in Europeans (8), which is not in high LD with rs820430 (*r*^2 ^=^ ^0.178 in CEU). Our study shows that rs13082711 also affects *SLC4A7* expression and reveals that it leads to altered NBCn1 protein levels in VSMCs and VECs which are cell types that play important roles in controlling BP. Importantly, our study reveals, for the first time, that the BP-associated genetic variant impacts on pH_*i*_ regulation, which has a direct and important implication in BP control.

It should be acknowledged that apart from VECs and VSMCs, there are other tissues such as the medullary thick ascending limb of the loop of Henle where *SLC4A7* may exert an effect on BP regulation. Additionally, data from the Genotype-Tissue Expression (GTEx) Project indicates that *SLC4A7* is also expressed in other tissues such as mammary tissue, transformed fibroblasts, prostate, lymphocytes, nerves and subcutaneous adipose tissue. Furthermore, the same database shows an association between the SNP rs13082711 and the expression level of the neighbouring gene, NEK10, in left ventricles. Possible effects on these other tissues were not explored in this study, but remain a potential parallel mechanism of action for the BP-associated SNP.

In summary, the work presented here has revealed that the BP-raising allele of rs13082711 is associated with allele-dependent DNA-nuclear protein interactions, greater *SLC4A7* transcript levels, higher NBCn1 protein levels and plasma membrane availability particularly in VSMCs, and increased rates of base uptake via Na^+^/${\text{HCO}}_{3}^{-}$ co-transport and higher resting pH_*i*_, once again more apparent in VSMCs. Notably, due to the large contribution of Na^+^/H^+ ^exchange to VEC pH_*i*_ regulation, the subtle genotypic effect is masked when Na^+^/H^+ ^exchange is present. This is not the case for VSMCs, where the genotypic effect persists even in the presence of active Na^+^/H^+ ^exchange. The missense variant Glu326Lys which is in high LD with rs13082711, had no significant effect on NBCn1 function, suggesting that the functional effect of the BP-associated variant is predominantly related to an influence on *SLC4A7* expression levels. The identification of a pathophysiological path from BP-associated genetic variation, to gene expression, and subsequently to gene function that alters cellular behaviour supports these genetic studies as a route to the discovery of drug targets.

## Materials and Methods

### Study samples

This study had ethical approval from Queen Mary, University of London (Protocol No.: Plaque-WHRI-01; NRES ref: 08/H0704/140, and subsequent amendments). Umbilical cords for cell isolation were obtained from the Royal London Hospital which serves the east London population where the two largest self-reported ethnic groups were Bangladeshi (32%) and White British (31%), with 21% of households being multi-ethnic. All tissue samples were fully anonymous before distribution to the recipient analysis groups, as per ethical approval. We derived primary cultures of human umbilical artery vascular smooth muscle cells (VSMCs) based on cell explants cultured on 0.2% w/v gelatin-coated polystyrene as established previously (41). We derived primary cultures of human umbilical vein endothelial cells (VECs) based on endoluminal collagenase digestion, cultured on 0.2% w/v gelatin-coated polystyrene as established previously (42).

The A10 rat thoracic aorta vascular smooth muscle cell line was obtained commercially from ATCC (CRL-1476) for overexpression studies. All cells were used before a maximal passage number of 6. Where indicated, serum-free media was used to induce synchronised cell arrest of VSMCs (27).

### Genotyping

DNA was extracted from cell culture samples (Qiagen, 69509) and genotyped in an array using the KASP™ method (Applied Bioscience). The primers used are listed in Supplementary Material, Table S2. The minor allele frequency of the study population (19.8%) approximates those in EUR (21.5%) and SAS (18.1%) in the 1000 Genomes Project samples.

### Allelic imbalance analyses

Total cellular RNA was isolated from cell culture samples (Macherey-Nagel, 740955), and reverse transcribed (Promega, M170). End-point RT-PCR was conducted using the primers listed in Supplementary Material, Table S3. The PCR products were size-separated via agarose gel electrophoresis, excised and cleaned up (Promega, A9281) before Sanger sequencing was conducted by the commercial service at the Genome Centre, Queen Mary University of London. The PeakPicker software (McGill University and Génome Québec Innovation Centre, California) was used to determine relative allele ratios from heterozygotes of interest (43).

### Formaldehyde-assisted isolation of regulatory elements

Formaldehyde-assisted isolation of regulatory elements (FAIRE) was conducted as adapted from the commercial kit from Millipore (#16-201). Briefly, formaldehyde was added to allow protein-DNA cross-linking, with glycine further added to quench the residual formaldehyde. The cells were lysed and the DNA sheared by a probe sonicator (Jencons Scientific, 690-024) for three pulses of 10 s, producing sheared DNA of lengths between 200 and 800 base pairs. The samples were de-crosslinked by heating, and the residual proteins were digested by proteinase K. The DNA fragments were then suitable for isolation by DNA extraction columns (Promega, A9281). The isolated chromatin-associated DNA was used for allelic imbalance analyses as described above.

### Electrophoretic mobility assay (EMSA) and DNA pulldown

Commercial complementary single-stranded oligonucleotides (either unlabelled or biotin-labelled) were annealed to form double-stranded oligonucleotides (see Supplementary Material, Table S4 for sequences). Nuclear extracts were isolated from VSMCs and VECs (Millipore, #2900). Both the nuclear extracts and double-stranded oligonucleotides were used for both EMSAs and DNA pulldown assays.

For EMSAs, the reaction mixture was combined with study-specific quantities of nuclear proteins, labelled antibody and unlabelled competitor antibodies. Unless where specified, quantities of nuclear proteins were 10 µg (2 µg/µl) and labelled oligonucleotides were 10 femtomoles (10^−^^14^ moles). When required for supershift assays, 1 or 5 ng of the antibody (rabbit anti-AP2γ IgG antibody, Santa Cruz, sc-8977 X; goat anti-PHOX2A IgG antibody, Santa Cruz, sc-13229 X; or rabbit anti-ZAC1 IgG antibody, Santa Cruz, sc-22811 X) was pre-incubated with the nuclear proteins before adding to the reactions. To account for the variable volumes of reactants, the final reaction volume was made up to 20 µl with nuclease-free water. The reaction mixture was electrophoresed on a 4% non-denaturing acrylamide gel, electrotransferred onto a nylon membrane (Amersham, RPN119B) and crosslinked with oven drying followed by 254 nm ultraviolet light at a dose of 0.12 J/cm^2^ (Biolink, BLX-254E). The biotin-labelled double-stranded oligonucleotides were detected by the LightShift® Chemiluminescent EMSA Kit (ThermoScientific, #89880).

DNA pulldown assays were conducted using streptavidin-conjugated agarose beads mixture (Sigma, S1638) based on manufacturer’s protocol as summarised below. For every reaction, 1 nmol of biotin-labelled double-stranded oligonucleotides were immobilised onto 50 µl of streptavidin-conjugated agarose beads mixture and washed. The bead-oligonucleotide complex was incubated with 500 µg of nuclear extract, and subsequently, the bead-oligonucleotide-protein complex was washed. The pulled-down proteins were eluted with 100 µl of 2x Laemmli buffer at 70^°^C. The eluate underwent SDS-PAGE gel electrophoresis and Ponceau S staining.

### Immunoblotting and subcellular fractionation

For immunoblotting, total cellular protein was isolated using RIPA buffer (50 mM Tris pH 7.4, 150 mM NaCl, 0.5% w/v sodium deoxycholate, 1% v/v NP-40, 0.1% w/v sodium dodecylsulfate) supplemented with protease inhibitors.

For subcellular fractionation by differential centrifugation, cells were homogenized with a homogenization buffer (10 mM Tris HCl pH 7.2, 1 mM EDTA pH 8.0, 250 mM sucrose) supplemented with protease inhibitor mixture. The cell homogenates were subjected to differential centrifugation with the supernatant removed at each step and resuspension in RIPA supplemented with protease inhibitors. The centrifugation sequences were: 900 g for 10 min (nuclear and cell debris fraction), 10,000 g for 5 min (mitochondrial/lysosomes/peroxisomes fraction) and 100,000 g for 60 min (membrane fraction). Each separate pellet was resuspended in RIPA buffer. The final supernatant was kept as the cytoplasmic fraction.

The samples derived from either method were electrophoresed using SDS-PAGE gels, electrotransferred to a PVDF membrane (Amersham, 10600021) and detected by enhanced chemiluminescence (Amersham, RPN2232). The antibodies used are listed in Supplementary Material, Table S5. Protease inhibitors reached the final concentration of 1 mM phenylmethanesulfonyl fluoride (Sigma, P7626), 2 µM leupeptin hemisulfate (Sigma, L2884), 1.5 µM pepstatin A (Sigma, P2465) and 0.15 µM aprotinin (Sigma, A1153) after addition to the samples.

### Intracellular pH (pH_*i*_) and Na^+^/${\text{HCO}}_{3}^{-}$-dependent pH_*i*_ recovery from intracellular acidosis

Experiments were conducted with cells cultured on 0.1% w/v poly-lysine (Sigma, P8920)-coated polystyrene flasks (BD-Falcon, #353107). Studies were conducted in a temperature-controlled stand and ambient air warmed to 37^°^C for 30 min before initiation of experiments. Ports were created to allow for aspiration or bubbling of gasses (5% CO_2_/95% air or 100% air) at a constant rate. Cells were incubated with 1 µM BCECF-AM (ThermoFisher, B-1170) for 20 min before being rinsed. A baseline *F_495_*/*F_440_* ratio was observed for 5 min before paired digital images at excitation wavelengths of 495 nm and 440 nm were obtained with exposure times of 800 and 1000 milliseconds respectively (Visitron Systems, Visiview Version 3), obtained every 3 s at periods of interest, otherwise at 15-s intervals. The initial resting baseline and final plateau was recorded for 1 min. A calibration curve was obtained at the end of the study where the cells are exposed to high-potassium buffers of varying pH with 5 mg/L nigericin (Sigma, N7143). These curves were highly reproducible, with coefficients of variation of <5%, and were near-linear in the pH_*i*_ section of interest (Supplementary Material, Fig. S10). For the constituents of buffers, see Supplementary Material, Table S6. The buffering capacity (mmol/L) was calculated based on the response to wash-out of NH_4_Cl using the formula β = Δ[${\text{NH}}_{4}^{\text{+}}$]_*i*_/ΔpH_*i*_. The [${\text{NH}}_{4}^{\text{+}}$]_*i*_ was calculated from the Henderson-Hasselbalch equation assuming equilibration of NH_3_ across the membrane and using a pK_a_ of 9.2, and ΔpH_*i*_ calculated by the difference in pH_*i*_ before and after washout of NH_4_Cl (18) The rate of net base uptake (mmol/L/min) during recovery from intracellular acidosis was calculated as the product of the rate of pH gain (ΔpH_*i*_ per minute calculated by linear regression over 15 s) and the buffering capacity (18).

### Cloning

The coding sequence of *SLC4A7* (Homo sapiens solute carrier family 4 member 7, transcript variant 1, mRNA Sequence ID: ref|NM_003615.4) was subcloned to pcDNA3.1(+) vector. Site-directed *in vitro* mutagenesis was conducted using the QuikChange II Site-Directed Mutagenesis Kit (Agilent, #200523), using the primer pair, forward: CCTGACGCTGACTC TCT TGG GAACTTCTGGAGG; reverse: CCTCCAGAAGTTCCCAAGA GAGT CAGCGTCAGG. PCR-driven overlap extension to generate the splice variant lacking Cassette II (Homo sapiens solute carrier family 4 member 7, transcript variant 3, mRNA Sequence ID: ref|NM_001258380.1) was based on the protocol described by Heckman and Pease (44), using the primer pair, forward: CTTGAAAGGAATGGTATTTTGGCCTCT; reverse: AGAGGCC AAA ATACCATTCCTTTCAAG. All plasmids had their sequences confirmed by Sanger sequencing.

### Cell culture and transfection

Transfections for A10 cells were conducted using liposome-based transfection with the X-tremeGENE reagent (Roche, 06366244001). For 10 cm^2^ surface area, ratios of 1 µg of plasmid, 200 µl of DMEM (Sigma, M4530) and 3 µl of the X-tremeGENE reagent were allowed to incubate for 20 min at room temperature before being added drop-wise into the culture surface already containing 2 ml of fresh media. The cells were then incubated in a humidified incubator kept at 37^°^C and 5% CO_2_ for 48 h without the need to replace the culture media prior to experiments.

### Statistical analyses

Comparisons between two independent groups were conducted using unpaired, two-tailed Student’s *t*-test or the Mann-Whitney *U*-test for parametric and non-parametric distributions, respectively. Comparisons between two paired groups were conducted using two-tailed, one-sample *t*-test or the Wilcoxon sign-ranked for parametric and non-parametric distributions, respectively. Predicted linear relationships were analyzed by least-squares linear regression and the derived slopes and y-axis intercepts compared. Comparisons between multiple paired groups were conducted using repeated-measures analysis of variance (ANOVA). Examination of the influence of two different independent variables (e.g. genotype and dose) on one dependent variable was conducted using two-way ANOVA. Graphical presentation and statistical analyses were conducted using Prism v5 (GraphPad Software). Values were expressed as mean ± standard error of the mean. A p-value of less than 0.05 was considered statistically significant. Multiple testing was further adjusted for by Bonferroni correction.

## Supplementary Material

Supplementary Material is available at *HMG* online.

*Conflict of Interest statement.* None declared.

## Supplementary Material

Supplementary Data

## References

[1] World Health Organisation. Global Health Risks: Mortality and burden of disease attributable to selected major risks. (2009) http://www.who.int/healthinfo/global_burden_disease/GlobalHealthRisks_report_full.pdf; date last accessed January 19, 2017.

[2] Gaziano T.A., Bitton A., Anand S., Weinstein M.C. International Society of Hypertension. (2009) The global cost of nonoptimal blood pressure. J. Hypertens., 27, 1472–1477. 19474763 10.1097/HJH.0b013e32832a9ba3

[3] Calhoun D.A., Jones D., Textor S., Goff D.C., Murphy T.P., Toto R.D., White A., Cushman W.C., White W., Sica D., (2008) Resistant hypertension: diagnosis, evaluation, and treatment. A scientific statement from the American Heart Association Professional Education Committee of the Council for High Blood Pressure Research. Hypertension, 51, 1403–1419. 18391085 10.1161/HYPERTENSIONAHA.108.189141

[4] Hong Y., de Faire U., Heller D.A., McClearn G.E., Pedersen N. (1994) Genetic and environmental influences on blood pressure in elderly twins. Hypertension, 24, 663–670. 7995622 10.1161/01.hyp.24.6.663

[5] Kupper N., Willemsen G., Riese H., Posthuma D., Boomsma D.I., de Geus E.J. (2005) Heritability of daytime ambulatory blood pressure in an extended twin design. Hypertension, 45, 80–85. 15557390 10.1161/01.HYP.0000149952.84391.54

[6] Levy D., Ehret G.B., Rice K., Verwoert G.C., Launer L.J., Dehghan A., Glazer N.L., Morrison A.C., Johnson A.D., Aspelund T., (2009) Genome-wide association study of blood pressure and hypertension. Nat. Genet., 41, 677–687. 19430479 10.1038/ng.384 PMC2998712

[7] Newton-Cheh C., Johnson T., Gateva V., Tobin M.D., Bochud M., Coin L., Najjar S.S., Zhao J.H., Heath S.C., Eyheramendy S., (2009) Genome-wide association study identifies eight loci associated with blood pressure. Nat. Genet., 41, 666–676. 19430483 10.1038/ng.361 PMC2891673

[8] International Consortium for Blood Pressure Genome-Wide Association Studies., Ehret G.B., Munroe P.B., Rice K.M., Bochud M., Johnson A.D., Chasman D.I., Smith A.V., Tobin M.D., Verwoert G.C., (2011) Genetic variants in novel pathways influence blood pressure and cardiovascular disease risk. Nature, 478, 103–109. 21909115 10.1038/nature10405 PMC3340926

[9] Kato N., Takeuchi F., Tabara Y., Kelly T.N., Go M.J., Sim X., Tay W.T., Chen C.H., Zhang Y., Yamamoto K., (2011) Meta-analysis of genome-wide association studies identifies common variants associated with blood pressure variation in east Asians. Nat. Genet., 43, 531–538. 21572416 10.1038/ng.834 PMC3158568

[10] Wain L.V., Verwoert G.C., O'Reilly P.F., Shi G., Johnson T., Johnson A.D., Bochud M., Rice K.M., Henneman P., Smith A.V., (2011) Genome-wide association study identifies six new loci influencing pulse pressure and mean arterial pressure. Nat. Genet., 43, 1005–1011. 21909110 10.1038/ng.922 PMC3445021

[11] Franceschini N., Fox E., Zhang Z., Edwards T.L., Nalls M.A., Sung Y.J., Tayo B.O., Sun Y.V., Gottesman O., Adeyemo A., (2013) Genome-wide association analysis of blood-pressure traits in African-ancestry individuals reveals common associated genes in African and non-African populations. Am. J. Hum. Genet., 93, 545–554. 23972371 10.1016/j.ajhg.2013.07.010 PMC3769920

[12] Ganesh S.K., Tragante V., Guo W., Guo Y., Lanktree M.B., Smith E.N., Johnson T., Castillo B.A., Barnard J., Baumert J., (2013) Loci influencing blood pressure identified using a cardiovascular gene-centric array. Hum. Mol. Genet., 22, 1663–1678. 23303523 10.1093/hmg/dds555 PMC3657476

[13] Tragante V., Barnes M.R., Ganesh S.K., Lanktree M.B., Guo W., Franceschini N., Smith E.N., Johnson T., Holmes M.V., Padmanabhan S., (2014) Gene-centric meta-analysis in 87,736 individuals of European ancestry identifies multiple blood-pressure-related loci. Am. J. Hum. Genet., 94, 349–360. 24560520 10.1016/j.ajhg.2013.12.016 PMC3951943

[14] Kato N., Loh M., Takeuchi F., Verweij N., Wang X., Zhang W., Kelly T.N., Saleheen D., Lehne B., Mateo Leach I., (2015) Trans-ancestry genome-wide association study identifies 12 genetic loci influencing blood pressure and implicates a role for DNA methylation. Nat. Genet., 47, 1282–1293. 26390057 10.1038/ng.3405 PMC4719169

[15] Choi I., Aalkjaer C., Boulpaep E.L., Boron W.F. (2000) An electroneutral sodium/bicarbonate cotransporter NBCn1 and associated sodium channel. Nature, 405, 571–575. 10850716 10.1038/35014615

[16] Damkier H.H., Nielsen S., Praetorius J. (2006) An anti-NH_2_-terminal antibody localizes NBCn1 to heart endothelia and skeletal and vascular smooth muscle cells. Am. J. Physiol. Heart Circ. Physiol., 290, H172–H180. 16126812 10.1152/ajpheart.00713.2005

[17] Boedtkjer E., Praetorius J., Füchtbauer E.M., Aalkjaer C. (2008) Antibody-independent localization of the electroneutral Na^+^-${\text{HCO}}_{3}^{-}$ cotransporter NBCn1 (slc4a7) in mice. Am. J. Physiol. Cell. Physiol., 294, C591–C603. 18077606 10.1152/ajpcell.00281.2007

[18] Boedtkjer E., Praetorius J., Aalkjaer C. (2006) NBCn1 (slc4a7) mediates the Na^+^-dependent bicarbonate transport important for regulation of intracellular pH in mouse vascular smooth muscle cells. Circ. Res., 98, 515–523. 16439691 10.1161/01.RES.0000204750.04971.76

[19] Boedtkjer E., Praetorius J., Matchkov V.V., Stankevicius E., Mogensen S., Füchtbauer A.C., Simonsen U., Füchtbauer E.M., Aalkjaer C. (2011) Disruption of Na^+^, ${\text{HCO}}_{3}^{-}$ cotransporter NBCn1 (slc4a7) inhibits NO-mediated vasorelaxation, smooth muscle Ca^2+^ sensitivity, and hypertension development in mice. Circulation, 124, 1819–1829. 21947296 10.1161/CIRCULATIONAHA.110.015974

[20] Vorum H., Kwon T.H., Fulton C., Simonsen B., Choi I., Boron W., Maunsbach A.B., Nielsen S., Aalkjaer C. (2000) Immunolocalization of electroneutral Na-${\text{HCO}}_{3}^{-}$ cotransporter in rat kidney. Am. J. Physiol. Renal Physiol., 279, F901–F909. 11053051 10.1152/ajprenal.2000.279.5.F901

[21] Damkier H.H., Nielsen S., Praetorius J. (2007) Molecular expression of SLC4-derived Na^+^-dependent anion transporters in selected human tissues. Am. J. Physiol. Regul. Integr. Comp. Physiol., 293, R2136–R2146. 17715183 10.1152/ajpregu.00356.2007

[22] Horie S., Yano S., Watanabe K. (1995) Intracellular alkalinization by NH4Cl increases cytosolic Ca^2+^ level and tension in the rat aortic smooth muscle. Life Sci., 56, 1835–1843. 7739357 10.1016/0024-3205(95)00155-y

[23] Boedtkjer E., Damkier H.H., Aalkjaer C. (2012) NHE1 knockout reduces blood pressure and arterial media/lumen ratio with no effect on resting pH*i* in the vascular wall. J. Physiol., 590, 1895–1906. 22351634 10.1113/jphysiol.2011.227132 PMC3573311

[24] Thomsen A.B., Kim S., Aalbaek F., Aalkjaer C., Boedtkjer E. (2014) Intracellular acidification alters myogenic responsiveness and vasomotion of mouse middle cerebral arteries. J. Cereb. Blood Flow Metab., 34, 161–168. 24192638 10.1038/jcbfm.2013.192 PMC3887363

[25] Fleming I., Hecker M., Busse R. (1994) Intracellular alkalinization induced by bradykinin sustains activation of the constitutive nitric oxide synthase in endothelial cells. Circ. Res., 74, 1220–1226. 7514511 10.1161/01.res.74.6.1220

[26] Cukiernik M., Hileeto D., Downey D., Evans T., Khan Z.A., Karmazyn M., Chakrabarti S. (2004) The role of the sodium hydrogen exchanger-1 in mediating diabetes-induced changes in the retina. Diabetes Metab. Res. Rev., 20, 61–71. 14737747 10.1002/dmrr.421

[27] Pardee A.B. (1974) A restriction point for control of normal animal cell proliferation. Proc. Natl Acad. Sci. USA, 71, 1286–1290. 4524638 10.1073/pnas.71.4.1286 PMC388211

[28] Grantham R. (1974) Amino acid difference formula to help explain protein evolution. Science, 185, 862–864. 4843792 10.1126/science.185.4154.862

[29] Boedtkjer E., Bunch L., Pedersen S.F. (2012) Physiology, pharmacology and pathophysiology of the pH regulatory transport proteins NHE1 and NBCn1: similarities, differences, and implications for cancer therapy. Curr. Pharm. Des., 18, 1345–1371. 22360557 10.2174/138161212799504830

[30] Danielsen A.A., Parker M.D., Lee S., Boron W.F., Aalkjaer C., Boedtkjer E. (2013) Splice cassette II of Na^+^,${\text{HCO}}_{3}^{-}$ cotransporter NBCn1 (slc4a7) interacts with calcineurin A: implications for transporter activity and intracellular pH control during rat artery contractions. J. Biol. Chem., 288, 8146–8155. 23382378 10.1074/jbc.M113.455386 PMC3605633

[31] Izzard A.S., Cragoe E.J. Jr., Heagerty A.M. (1991) Intracellular pH in human resistance arteries in essential hypertension. Hypertension, 17, 780–786. 1646163 10.1161/01.hyp.17.6.780

[32] Aalkjaer C., Cragoe E.J. Jr. (1988) Intracellular pH regulation in resting and contracting segments of rat mesenteric resistance vessels. J. Physiol., 402, 391–410. 2976824 10.1113/jphysiol.1988.sp017211 PMC1191898

[33] Wu S., Song T., Zhou S., Liu Y., Chen G., Huang N., Liu L. (2008) Involvement of Na^+^/H^+^ exchanger 1 in advanced glycation end products-induced proliferation of vascular smooth muscle cell. Biochem. Biophys. Res. Commun., 375, 384–389. 18703017 10.1016/j.bbrc.2008.08.008

[34] Brenninkmeijer L., Kuehl C., Geldart A.M., Arons E., Christou H. (2011) Heme oxygenase-1 does not mediate the effects of extracellular acidosis on vascular smooth muscle cell proliferation, migration, and susceptibility to apoptosis. J. Vasc. Res., 48, 285–296. 21273783 10.1159/000321555

[35] Boedtkjer E., Bentzon J.F., Dam V.S., Aalkjaer C. (2016) Na^+^, ${\text{HCO}}_{3}^{-}$-cotransporter NBCn1 increases pH_i_ gradients, filopodia, and migration of smooth muscle cells and promotes arterial remodelling. Cardiovasc. Res., 111, 227–239. 27076468 10.1093/cvr/cvw079

[36] Hatori N., Fine B.P., Nakamura A., Cragoe E. Jr., Aviv A. (1987) Angiotensin II effect on cytosolic pH in cultured rat vascular smooth muscle cells. J. Biol. Chem., 262, 5073–5078. 3031039

[37] Touyz R.M., Schiffrin E.L. (1993) Effects of angiotensin II and endothelin-1 on platelet aggregation and cytosolic pH and free Ca^2+^ concentrations in essential hypertension. Hypertension, 22, 853–862. 8244517 10.1161/01.hyp.22.6.853

[38] Austin C., Wray S. (1993) Changes of intracellular pH in rat mesenteric vascular smooth muscle with high-K depolarization. J. Physiol., 469, 1–10. 8271193 10.1113/jphysiol.1993.sp019800 PMC1143857

[39] Lu X., Wang L., Lin X., Huang J., Charles Gu C., He M., Shen H., He J., Zhu J., Li H., (2015) Genome-wide association study in Chinese identifies novel loci for blood pressure and hypertension. Hum. Mol. Genet., 24, 865–874. 25249183 10.1093/hmg/ddu478 PMC4303798

[40] Wang L., Li H., Yang B., Guo L., Han X., Li L., Li M., Huang J., Gu D. (2016) The hypertension risk variant rs820430 functions as an enhancer of *SLC4A7*. Am. J. Hypertens., pii: hpw127. 10.1093/ajh/hpw127 27784683

[41] Leik C.E., Willey A., Graham M.F., Walsh S.W. (2004) Isolation and culture of arterial smooth muscle cells from human placenta. Hypertension, 43, 837–840. 14967841 10.1161/01.HYP.0000119191.33112.9c

[42] Jaffe E.A., Nachman R.L., Becker C.G., Minick C.R. (1973) Culture of human endothelial cells derived from umbilical veins. Identification by morphologic and immunologic criteria. J. Clin. Invest., 52, 2745–2756. 4355998 10.1172/JCI107470 PMC302542

[43] Ge B., Gurd S., Gaudin T., Dore C., Lepage P., Harmsen E., Hudson T.J., Pastinen T. (2005) Survey of allelic expression using EST mining. Genome Res., 15, 1584–1591. 16251468 10.1101/gr.4023805 PMC1310646

[44] Heckman K.L., Pease L.R. (2007) Gene splicing and mutagenesis by PCR-driven overlap extension. Nat. Protoc., 2, 924–932. 17446874 10.1038/nprot.2007.132

