# Supplementary material for: Increased NBCn1 expression, Na+/HCO3− co-transport and intracellular pH in human vascular smooth muscle cells with a risk allele for hypertension
Source: Hum Mol Genet. 2017 Jan 13;26(5):989–1002. doi: 10.1093/hmg/ddx015 (PMC5409084; doi:10.1093/hmg/ddx015)
Supplement: Supplementary Data [file ddx015_Supp.docx]

**Supplementary Figure 1**


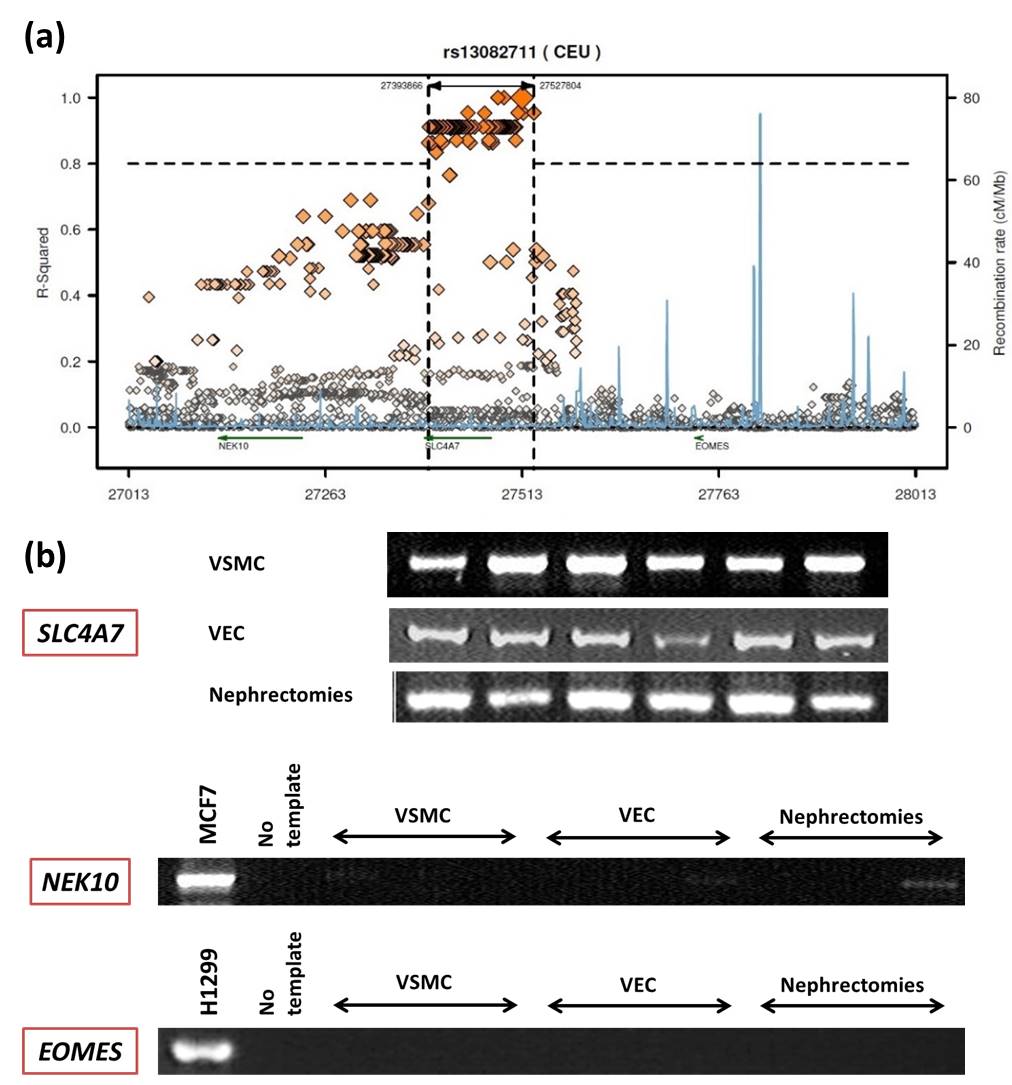


**Supplementary Figure 2**


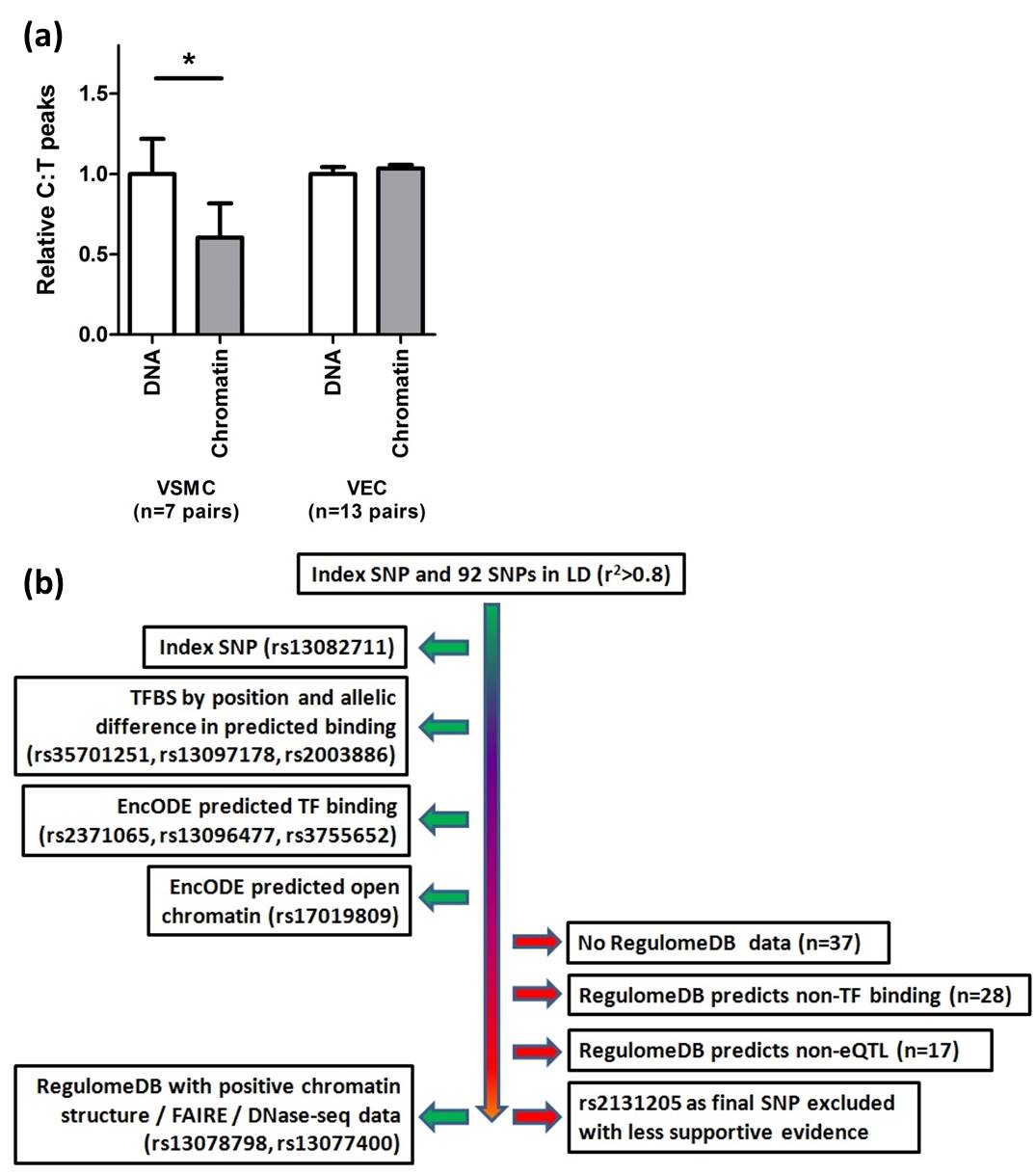


**Supplementary Figure 3**


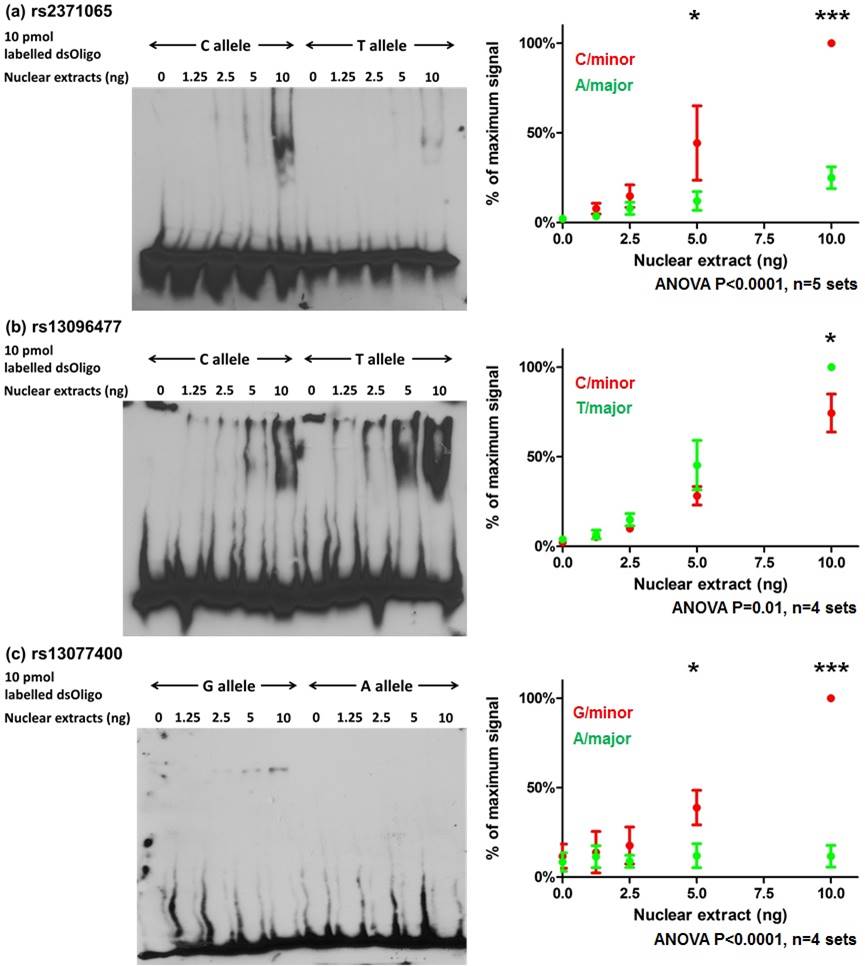


**Supplementary Figure 4**


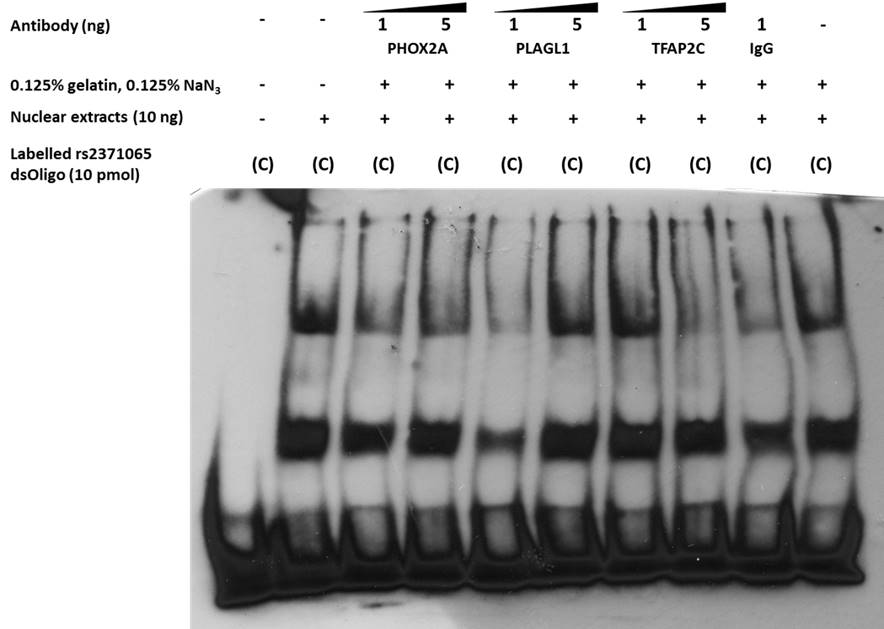


**Supplementary Figure 5**


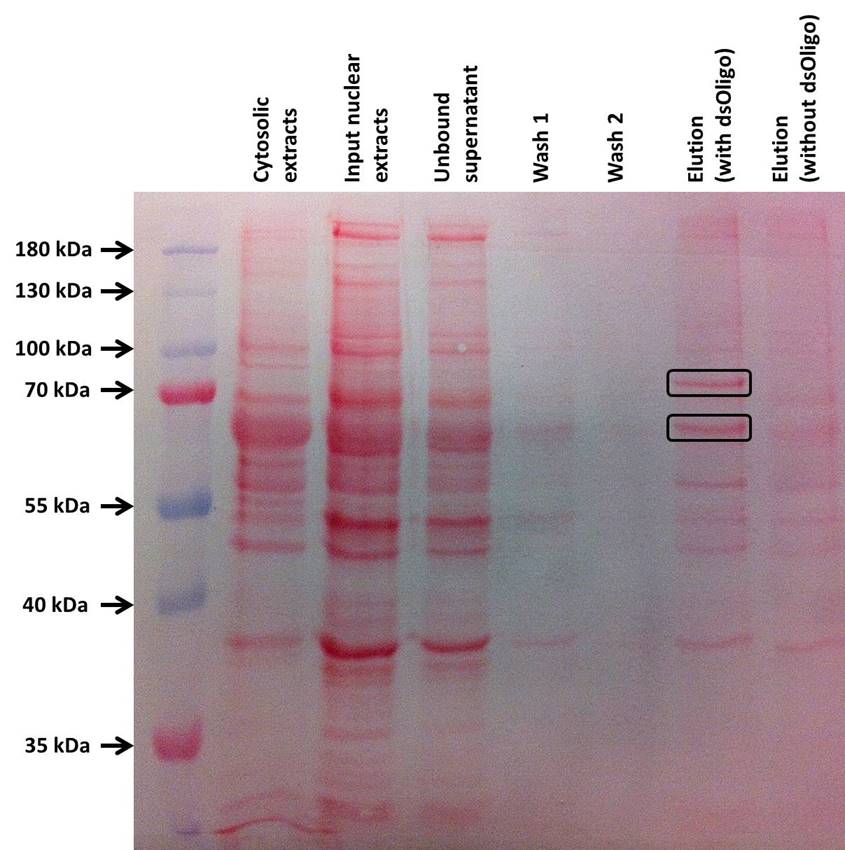


**Supplementary Figure 6**


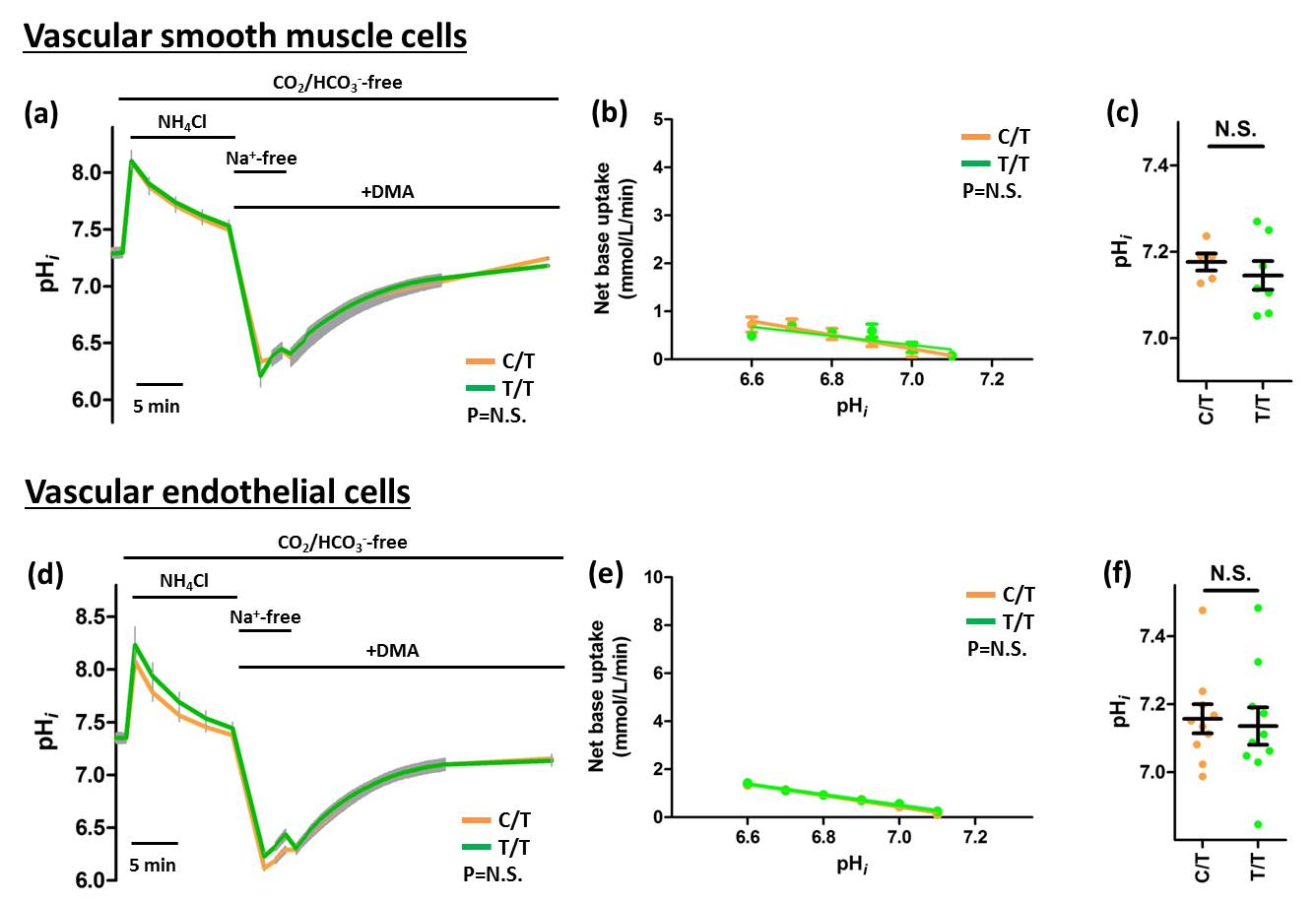


**Supplementary Figure 7**


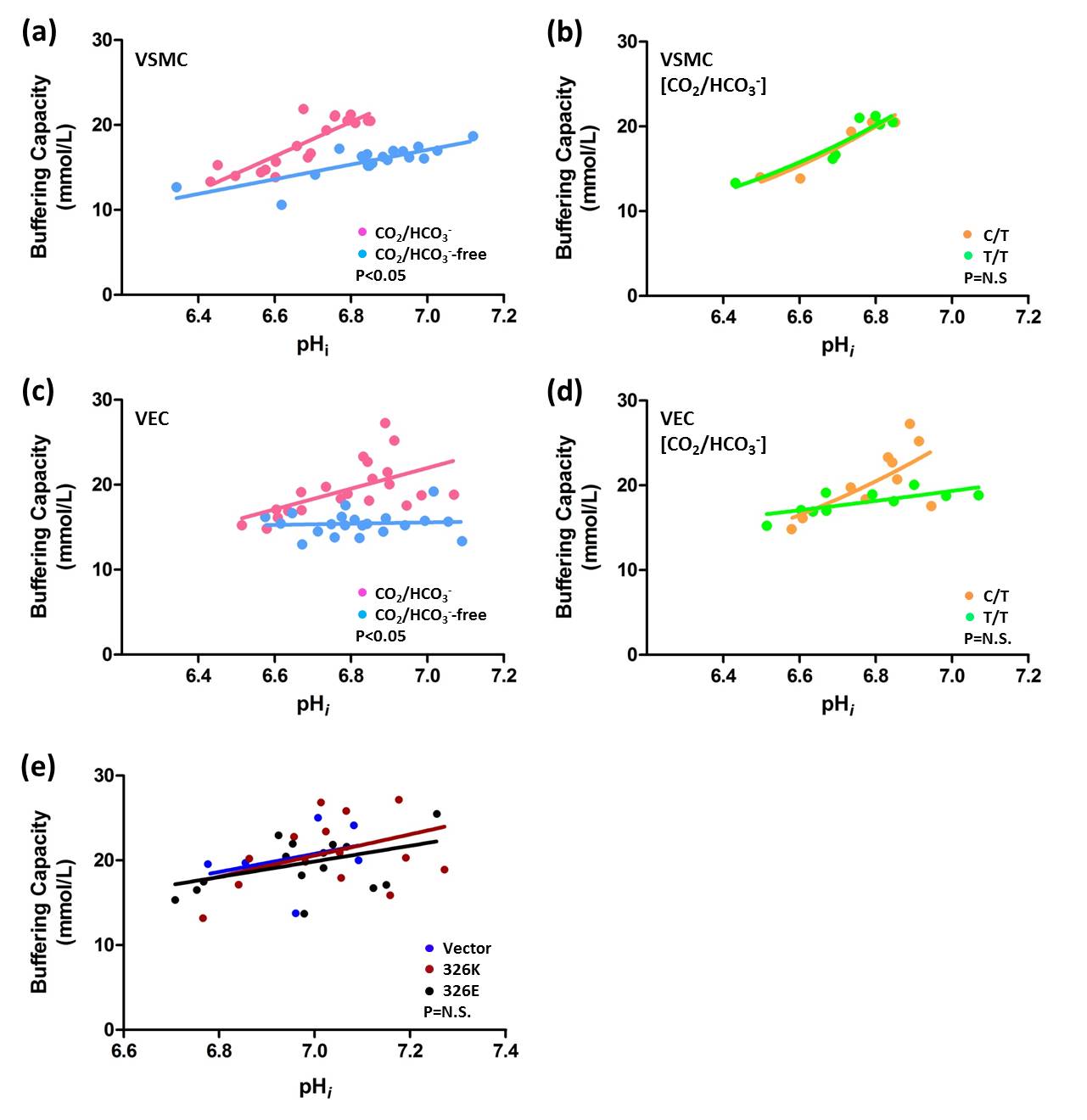


**Supplementary Figure 8**

**
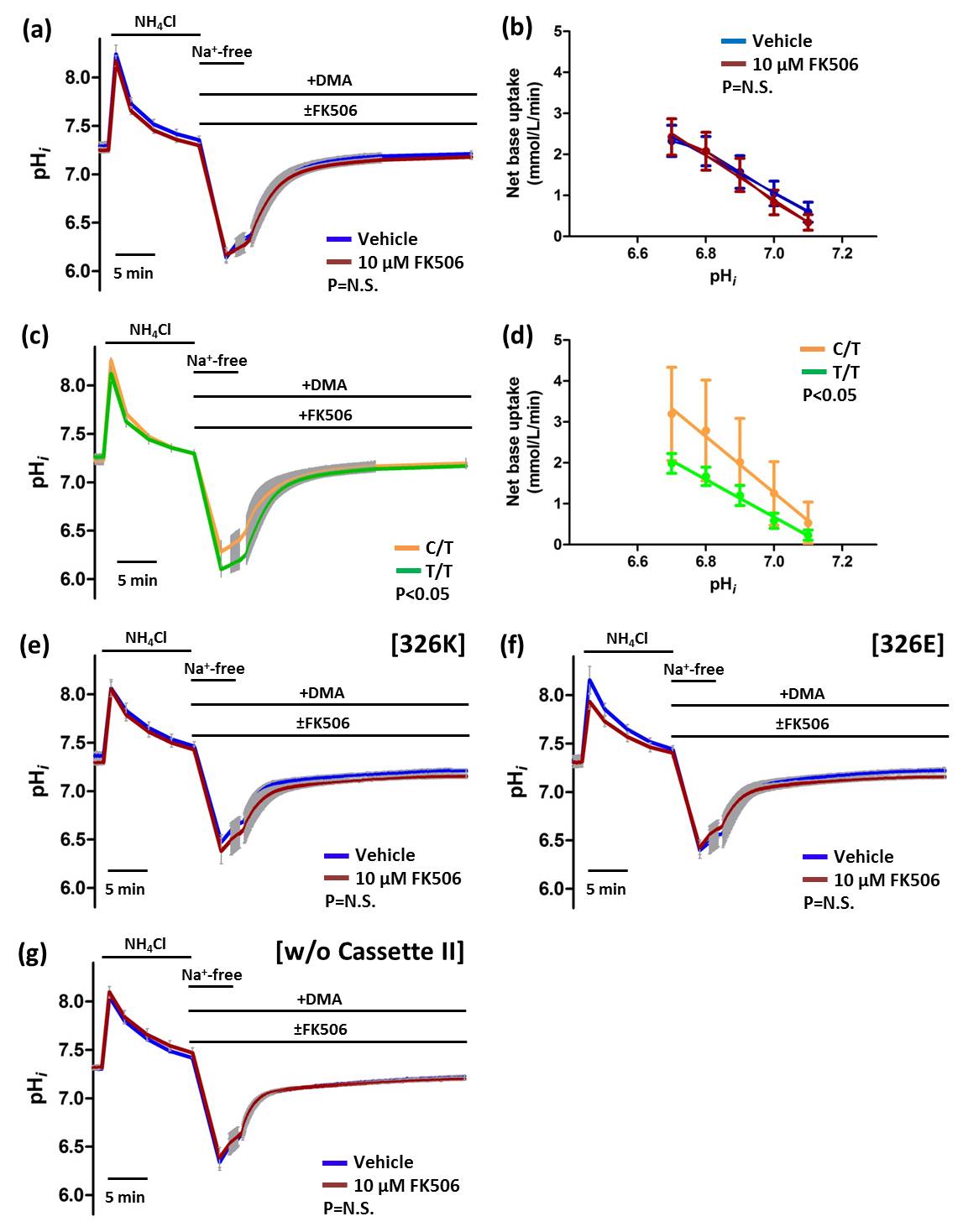
**

**Supplementary Figure 9**


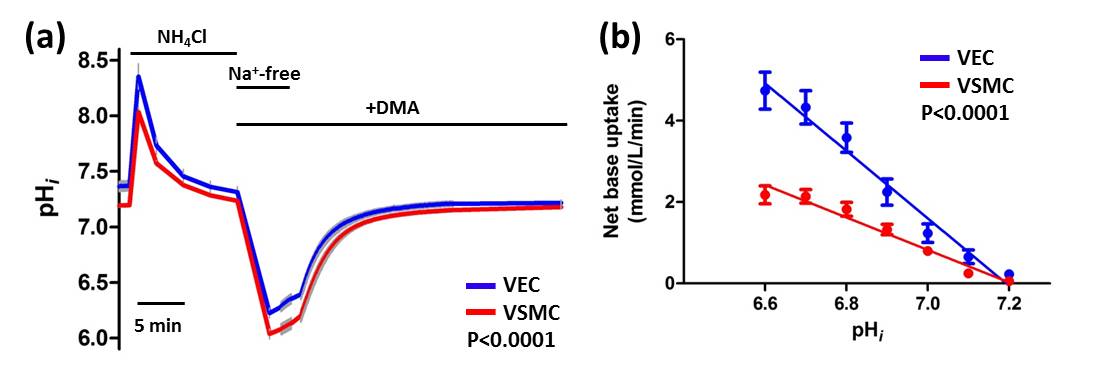


**Supplementary Figure 10**


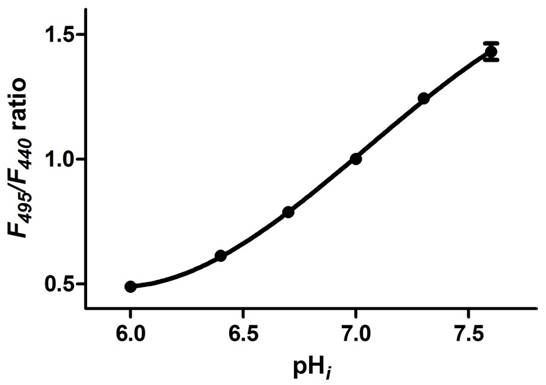


**Supplementary Table 1**

| **Prediction tool** | **Reference** | **Prediction (Score)** |
| --- | --- | --- |
| **Mutation Assessor** | http://mutationassessor.org/ | Neutral (0.145) |
| **MutPred** | http://mutpred.mutdb.org/ | No hypothesis (0.332). |
| **PHD-SNP** | http://gpcr2.biocomp.unibo.it/~emidio/PhD-SNP/PhD-SNP.htm | Neutral (8) |
| **PolyPhen2** | http://genetics.bwh.harvard.edu/pph2/ | Benign |
| **SIFT** | http://sift.jcvi.org/www/SIFT_enst_submit.html | Tolerated (0.87) |
| **SNAP** | https://www.rostlab.org/services/SNAP | Minimal effect (2) |
| **SNP&GO** | http://snps-and-go.biocomp.unibo.it/snps-and-go/ | Neutral (6) |

**Supplementary Table 1** Predicted functional impact of the E326K polymorphism by online tools. Online prediction tools listed in alphabetical order. Note that the scales for scores are different for all the separate prediction tools.

**Supplementary Table 2**

| **KASPar Genotyping Primers** | | |
| --- | --- | --- |
| **SNP** | **Primer type (reporter)** | **Sequence** |
| **rs13082711** | C allele (VIC) | GAAGGTCGGAGTCAACGGATTAAGATCTGTTTAAAAACAGCTCCCTAC |
|  | T allele (FAM) | GAAGGTGACCAAGTTCATGCTTAAAGATCTGTTTAAAAACAGCTCCCTAT |
|  | Common primer | AGTTTAGCAAAGCTACAGAACAAGGACAT |
| **rs13096477** | C allele (VIC) | GAAGGTCGGAGTCAACGGATTACACAAACATCACCACCGGAGG |
|  | T allele (FAM) | GAAGGTGACCAAGTTCATGCTGACACAAACATCACCACCGGAGA |
|  | Common primer | CCCGTCCATCTTCTTTATCTGATTCTTTA |
| **rs3755652** | C allele (VIC) | GAAGGTCGGAGTCAACGGATTCTGACCTCCAGAAGTTCCCAAG |
|  | T allele (FAM) | GAAGGTGACCAAGTTCATGCTCCTGACCTCCAGAAGTTCCCAAA |
|  | Common primer | TCACTGGCAGGTGAAACCAGTAGTT |

**Supplementary Table 2** List of KASPar genotyping primer sequences

**Supplementary Table 3**

| **Assay** |  | **Sequence** | **Annealing temp / Mg^2+^ conc** | **Amplicon size (bp)** |
| --- | --- | --- | --- | --- |
| **rs13096477 genomic DNA** | **Forward** | CCCAATACAATACATTCCCCATAC | 57 ^o^C /  2 mM | 459 |
|  | **Reverse** | TGGGCACAATATTCTGTGTAGTG |  |  |
| **rs13096477 complementary DNA** | **Forward** | CCTGGTCCTGATGAAGAAGC | 57 ^o^C /  2 mM | 406 |
|  | **Reverse** | CGCCATCTTCAACATCCTCT |  |  |
| ***SLC4A7* splice**  **(all three primers in a single PCR)** | **Forward** | CTGGCCAATTAGACGAGTCC(Exon 6) | 57 ^o^C /  2 mM | Variable |
|  | **Rev 1** | TCAGCACCCGTAGGAATTTT(Exon 7) |  |  |
|  | **Rev 2** | CGCTGACTCTTTTGGGAACT(Exon 8) |  |  |
| ***SLC4A4* cDNA** | **Forward** | GGCTTCTTCTCTCCCACAGT | 57 ^o^C /  2 mM | 129 |
|  | **Reverse** | TTCTTGGTTTGATGCCGGTG |  |  |
| ***SLC9A1* cDNA** | **Forward** | cggttctggctgtctttgag | 57 ^o^C /  2 mM | 181 |
|  | **Reverse** | accacgaagaagctcaggaa |  |  |
| **β-actin cDNA** | **Forward** | TTCTACAATGAGCTGCGTGTG | 57 ^o^C /  2 mM | 122 |
|  | **Reverse** | GGGGTGTTGAAGGTCTCAAA |  |  |
| ***EOMES* cDNA** | **Forward** | CCACTGCCCACTACAATGTG | 55 ^o^C /  3 mM | 480 |
|  | **Reverse** | GACCTCCAGGGACAATCTGA |  |  |
| ***NEK10* cDNA** | **Forward** | AATGGCCAATGAAGCTTTTG | 57 ^o^C /  2 mM | 377 |
|  | **Reverse** | TACGCTGAGGATGTTTGCTG |  |  |

**Supplementary Table 3** List of end-point PCR and RT-PCR primer sequences

**Supplementary Table 4**

| **SNP** | **Allele** |  | **Sequence** |
| --- | --- | --- | --- |
| **rs13077400** | **A** | **Forward** | GAAGATGACCAAGAAGAAGAG |
|  |  | **Reverse** | CTCTTCTTCTTGGTCATCTTC |
|  | **G** | **Forward** | GAAGATGACCGAGAAGAAGAG |
|  |  | **Reverse** | CTCTTCTTCTCGGTCATCTTC |
| **rs13078798** | **A** | **Forward** | CAACCTCACCAATGGAAACTG |
|  |  | **Reverse** | CAGTTTCCATTGGTGAGGTTG |
|  | **G** | **Forward** | CAACCTCACCGATGGAAACTG |
|  |  | **Reverse** | CAGTTTCCATCGGTGAGGTTG |
| **rs13082711** | **C** | **Forward** | CAGCTCCCTACGATGGGGGCC |
|  |  | **Reverse** | GGCCCCCATCGTAGGGAGCTG |
|  | **T** | **Forward** | CAGCTCCCTATGATGGGGGCC |
|  |  | **Reverse** | GGCCCCCATCATAGGGAGCTG |
| **rs13096477** | **C** | **Forward** | TATCTTTTCTCCTCCGGTGGT |
|  |  | **Reverse** | ACCACCGGAGGAGAAAAGATA |
|  | **T** | **Forward** | TATCTTTTCTTCTCCGGTGGT |
|  |  | **Reverse** | ACCACCGGAGAAGAAAAGATA |
| **rs1309717** | **C** | **Forward** | CTGCAACATCCGGAAAGAGTC |
|  |  | **Reverse** | GACTCTTTCCGGATGTTGCAG |
|  | **T** | **Forward** | CTGCAACATCTGGAAAGAGTC |
|  |  | **Reverse** | GACTCTTTCCAGATGTTGCAG |
| **rs17019809** | **A** | **Forward** | CCCGTAAAACAAATGGAAACC |
|  |  | **Reverse** | GGTTTCCATTTGTTTTACGGG |
|  | **G** | **Forward** | CCCGTAAAACGAATGGAAACC |
|  |  | **Reverse** | GGTTTCCATTCGTTTTACGGG |
| **rs2003886** | **C** | **Forward** | TGCGTCTCTGCGTCTGCAGCT |
|  |  | **Reverse** | AGCTGCAGACGCAGAGACGCA |
|  | **T** | **Forward** | TGCGTCTCTGTGTCTGCAGCT |
|  |  | **Reverse** | AGCTGCAGACACAGAGACGCA |
| **rs2371065** | **A** | **Forward** | TCCCACCTTCATAATTTTGTT |
|  |  | **Reverse** | AACAAAATTATGAAGGTGGGA |
|  | **C** | **Forward** | TCCCACCTTCCTAATTTTGTT |
|  |  | **Reverse** | AACAAAATTAGGAAGGTGGGA |
| **rs35701251** | **A** | **Forward** | TTCAGAATACATCCTGAGTAT |
|  |  | **Reverse** | ATACTCAGGATGTATTCTGAA |
|  | **T** | **Forward** | TTCAGAATACTTCCTGAGTAT |
|  |  | **Reverse** | ATACTCAGGAAGTATTCTGAA |
| **rs3755652** | **C** | **Forward** | CGCTGACTCTCTTGGGAACTT |
|  |  | **Reverse** | AAGTTCCCAAGAGAGTCAGCG |
|  | **T** | **Forward** | CGCTGACTCTTTTGGGAACTT |
|  |  | **Reverse** | AAGTTCCCAAAAGAGTCAGCG |

**Supplementary Table 4** List of EMSA oligonucleotide sequences

**Supplementary Table 5**

| **Name** | **Manufacturer** | **Product No** | **Working concentration (μg/ml)** |
| --- | --- | --- | --- |
| **Rabbit Anti-SLC4A7 IgG antibody** | Abcam | ab82335 | 1 |
| **Horse anti-mouse IgG, HRP-linked Antibody** | New England Biolabs | 7076S | 0.1 |
| **Goat anti-rabbit IgG, HRP-linked Antibody** | New England Biolabs | 7074S | 0.13 |
| **Mouse Anti-GAPDH IgG antibody** | Santa Cruz | sc-47724 | 0.1 |
| **Mouse Anti-α Tubulin IgM antibody** | Santa Cruz | sc-53646 | 1 |
| **Mouse IgG anti-human VE-Cadherin** | Abcam | ab7047 | 1 |
| **Mouse IgG anti-human N-Cadherin** | Abcam | ab18203 | 1 |
| **Donkey anti-rabbit IgG - H&L Alexa Fluor® 594** | Abcam | ab150076 | 2 |
| **Goat anti-mouse IgG - H&L Alexa Fluor® 488** | Abcam | ab150113 | 2 |

**Supplementary Table 5** List of antibodies

**Supplementary Table 6**

| **Solution** | **Final concentration** |
| --- | --- |
| **Physiological saline solution**  **(PSS)**  **(titrated to pH 7.45)** | 141 mM Na^+^  122 mM Cl^-^  25 mM HCO_3_^-^  10 mM HEPES  5.5 mM glucose  4 mM K^+^  1.6 mM Ca^2+^  1.2 mM Mg^2+^  1.2 mM SO_4_^2-^  1.18 mM H_2_PO_4_^-^  0.03 mM EDTA |
| **Sodium-free physiological saline solution**  **(-Na^+^ PSS)**  **(titrated to pH 7.45)** | 116 mM NMDG^+^  25 mM choline^+^  122 mM Cl^-^ (in theory after titrating pH with HCl)  25 mM HCO_3_^-^  10 mM HEPES  5.5 mM glucose  4 mM K^+^  1.6 mM Ca^2+^  1.2 mM Mg^2+^  1.2 mM SO_4_^2-^  1.18 mM H_2_PO_4_^-^  0.03 mM EDTA |
| **Bicarbonate-free physiological saline solution**  **(-HCO_3_^-^ PSS)**  **(titrated to pH 7.45)** | 141 mM Na^+^  147 mM Cl^-^  10 mM HEPES  5.5 mM glucose  4 mM K^+^  1.6 mM Ca^2+^  1.2 mM Mg^2+^  1.2 mM SO_4_^2-^  1.18 mM H_2_PO_4_^-^  0.03 mM EDTA |
| **Sodium- and bicarbonate-free physiological saline solution**  **(-Na^+^/-HCO_3_^-^ PSS)**  **(titrated to pH 7.45)** | 141 mM NMDG^+^  147 mM Cl^-^ (in theory after titrating pH with HCl)  10 mM HEPES  5.5 mM glucose  4 mM K^+^  1.6 mM Ca^2+^  1.2 mM Mg^2+^  1.2 mM SO_4_^2-^  1.18 mM H_2_PO_4_^-^  0.03 mM EDTA |
| **Sodium-free, high-potassium physiological saline solution**  **(-HCO_3_^-^/High-K^+^ PSS)**  **(titrated to specified pH)** | 68.8 mM NMDG^+^  25 mM choline^+^  122 mM Cl^-^  25 mM HCO_3_^-^  10 mM HEPES  5.5 mM glucose  51.2 mM K^+^  1.6 mM Ca^2+^  1.2 mM Mg^2+^  1.2 mM SO_4_^2-^  1.18 mM H_2_PO_4_^-^  0.03 mM EDTA |

**Supplementary Table 6** List of buffer components for intracellular pH studies
